# Estimated cost-savings from integrated care for HIV, diabetes and hypertension in sub-Saharan Africa: a cost-minimisation analysis

**DOI:** 10.1080/16549716.2025.2556364

**Published:** 2025-09-09

**Authors:** Gerard Joseph Abou Jaoude, Sokoine Kivuyo, Josephine Birungi, Joseph Okebe, Kaushik Ramaiya, Ivan Namakoola, Simple Ouma, Anupam Garrib, Erik van Widenfelt, Jeffrey Victor Lazarus, Gerald Mutungi, Omary Ubuguyu, Mina Nakawuka Ssali, Nelson K Sewankambo, Jolene Skordis, Sayoki Mfinanga, Moffat J Nyirenda, Shabbar Jaffar, Neha Batura

**Affiliations:** a Institute for Global Health, University College London, London, UK; b Muhimbili Centre, National Institute for Medical Research, Dar es Salaam, Tanzania; c Barcelona Institute for Global Health (ISGlobal) Hospital Clínic, University of Barcelona, Barcelona, Spain; d The AIDS Support Organisation, Mulago Hospital Complex, Kampala, Uganda; e Medical Research Council/Uganda Virus Research Institute & London School of Hygiene and Tropical Medicine (MRC/UVRI & LSHTM), Uganda Research Unit, Entebbe, Uganda; f Tanzania NCDs Alliance, Dar es Salaam, Tanzania; g Shree Hindu Mandal Hospital, Dar es Salaam, Tanzania; h Non-Communicable Diseases Control Programme, Ministry of Health, Kampala, Uganda; i Ministry of Health, United Republic of Tanzania Ministry of Health, Dodoma, Tanzania; j AIDS Control Programme, Ministry of Health, Kampala, Uganda; k School of Public Health, Makerere University College of Health Sciences, Kampala, Uganda

**Keywords:** Economic evaluation, integrated care, randomised trial, non-communicable, chronic care

## Abstract

**Background:**

In a cluster-randomised trial in Uganda and Tanzania, we showed that integrated management, compared with standard vertical care, could achieve a high standard of care for diabetes and hypertension without adversely affecting outcomes for HIV. However, evidence on the value for money of integrated care is needed to inform policy.

**Objective:**

Our economic evaluation aimed to establish the value for money of integrated care compared with vertical care for HIV, diabetes and hypertension.

**Methods:**

A societal perspective was adopted, considering provider and patient costs for integrated and standard care in Uganda and Tanzania over one year. Provider costs were estimated for 6714 participants based on five representative health facilities per country. Patient costs were captured via questionnaire from a sub-sample of 2708 participants. Provider costs at scale were estimated using national prevalence and utilisation data. Key inputs were varied in two-way sensitivity analyses.

**Results:**

Among participants with single conditions, mean provider and patient costs per patient-visit did not differ significantly between integrated and standard care. Among participants with multiple conditions, mean provider and patient costs per patient-visit were, respectively, Int$18.67 (95%CI 10.89–26.45, *p* < 0.0001) and Int$5.86 (95%CI 2.57–9.16, *p* = 0.0005) lower in integrated clinics. If scaled up, integrated care could save providers Int$229 million in Uganda and Int$72 million in Tanzania. From a societal perspective, integrated care for participants with multiple conditions generated mean cost-savings of Int$41.54 (95%CI 29.42–53.67, *p* < 0.0001) per patient-visit.

**Conclusion:**

Integrated care is cost-saving from a societal perspective and should be considered for scale-up in Tanzania, Uganda and similar settings.

**Trial registration number:**

ISRCTN43896688

## Paper Context

- **Main findings**: Among people living with multiple health conditions in Tanzania and Uganda, the integrated model of care for HIV, hypertension and diabetes is significantly cost-saving for both providers and patients, when compared with standard care.
- **Added knowledge**: To the best of our knowledge, our study is the first societal economic evaluation to evaluate the value for money and costs at scale of integrated versus standard care for HIV, diabetes and hypertension within a randomised-controlled trial in sub-Saharan Africa.
- **Global health impact for policy and action**: Findings from our study provide a strong case for policymakers in Tanzania and Uganda, as well as similarly resource constrained settings in sub-Saharan Africa and beyond, on the value for money of scaling up integrated care for HIV, diabetes and hypertension.

## Background

Care for HIV in sub-Saharan Africa, and for other chronic conditions such as diabetes and hypertension, is delivered through separate (vertical) clinics [1]. Diabetes care is sometimes only available at secondary or higher-level facilities. Vertical care reorients health systems to treat health conditions separately, rather than take a holistic approach towards the health needs of the patient. This also impedes the strengthening of primary health care [2] and leads to the duplication of services. Given the increasing rate of multiple chronic health conditions [3], the negative impacts and inefficiencies of vertical care will likely become more acute over time.

HIV and non-communicable diseases (NCDs) already absorb a substantial portion of national and household-level financial resources in sub-Saharan Africa. For example, in 2019, combined spending on HIV and NCDs was equivalent to 2% of gross domestic product (GDP) in Tanzania and Uganda, or 41% and 51% of national health expenditure, respectively [4]. While donor funding accounted for 75% of total spending on HIV in Tanzania and 98% in Uganda, patients bore a substantial share of the financial burden related to NCDs – with private household spending accounting for 28% of total health spending on NCDs in Tanzania and 81% in Uganda [4]. In addition, donor funding is no longer increasing, risks being withdrawn, and there are growing calls to expand domestic health financing [5]. In practice, however, government health expenditure rarely replaces decreases in donor funding [6].

Considering increasing demands for healthcare from rising rates of multimorbidity, yet limited government funding and human resources for health, there is an urgent need to consider a reorganisation of health systems in sub-Saharan Africa from vertical to potentially more efficient and cost-effective integrated models of care [5]. Studies evaluating integrated models of care in sub-Saharan countries have reported high retention in care and no worsening of key clinical outcomes [7,8]. However, evidence is required to inform policymakers of the likely value for money of transitioning from standard care to integrated care in resource constrained settings [9].

There remains insufficient evidence on the costs and value for money of integrated models of care, despite the importance of such evidence in informing effective decision-making. One study found that integrated care for HIV, diabetes and hypertension likely reduces health service and household costs [7]. However, this study was a single-arm cohort study that did not compare the costs and effectiveness of receiving integrated care with standard vertical care. A follow-up to the single-arm cohort study using a cluster randomised-controlled design found that integrated care is likely cost-saving to health providers when compared with standard vertical care [8]. However, no study has yet assessed the cost and value of integrated care for HIV, diabetes and hypertension for both providers and patients within a randomised-controlled trial in Tanzania, Uganda or other comparable settings.

This paper fills this evidence gap by estimating the societal costs and savings attributable to integrated care, compared with standard care for HIV, diabetes and hypertension as part of a pragmatic parallel arm cluster randomised-controlled trial in Tanzania and Uganda [8]. To our knowledge, this is the first societal economic evaluation of integrated care for HIV, diabetes and hypertension to be conducted in sub-Saharan countries. Results from this study can inform the prioritisation of integrated care models in low- and middle-income countries in sub-Saharan Africa.

## Methods

### INTE-AFRICA trial

The INTE-AFRICA trial has been described in detail elsewhere [1,8]. Briefly, INTE-AFRICA was a pragmatic parallel arm cluster randomised-controlled trial that compared the provision of integrated care with standard, vertical care for people aged 18 years or over living with HIV, diabetes and/or hypertension in Tanzania and Uganda. A total of 32 medium-large health facilities participated in the trial, with 15 facilities in and around Dar es Salaam, Tanzania and 17 in Kampala, Uganda. All selected facilities provided primary care, were led by medical officers, and had a minimum of 100 people living with diabetes regularly accessing care. Patients participating in INTE-AFRICA were managed by government health staff and facilities operated close to normal health service conditions.

### Outcomes

The trial had two co-primary endpoints: (1) retention in care for participants with diabetes or hypertension and (2) plasma viral load suppression for those with HIV [8]. Retention in care was defined as attending a clinic within the last 6 months of follow-up and was tested for superiority between trial arms. Plasma viral load suppression for people living with HIV ( > 1000 copies per mL) was tested for non-inferiority between trial arms. As detailed further in the main trial paper [8], generalised estimating equations were used to analyse co-primary trial outcomes in the intention-to-treat population. By endline, retention in care was found to be similarly high in both arms, along with equivalent levels of viral load suppression. Given that the main trial analysis has already established similar co-primary outcomes between trial arms [8], the economic evaluation presented in this paper focuses on establishing whether costs differ between integrated and standard care arms, assuming that both approaches yield statistically indistinguishable outcomes [10,11]. As such, outcomes are held constant between arms.

### Facility-based vertical and integrated care

Vertical care, i.e standard care or treatment as usual in both Uganda and Tanzania, means a patient seeking care for HIV receives care at the HIV clinic and a patient seeking care for hypertension or diabetes would receive care at those respective clinics. In practice, this involves separate waiting areas, separate clinicians and lab technicians, and the collection of medications at different places or times. Patients can also receive care on completely different days as clinics for particular conditions may only run on specific days. Less prevalent conditions may only have a clinic once a week. By comparison, integrated care in INTE-AFRICA, involved the management of HIV, hypertension and diabetes at a single site, i.e. an integrated clinic. Integrated clinics involved shared registration and waiting areas, clinicians and lab technicians, pharmacies and medical storage areas [8].

### Economic evaluation objective

We conducted a within-trial economic evaluation to compare the cost of integrated care with standard care from a societal perspective [12] (combined costs to providers, participants and any household caregivers for children of participants receiving care) and the likely total cost of implementation at scale. We calculated economic provider, patient and household caregiver costs over a 12-month time horizon, in line with the duration of study implementation and follow-up. A brief description of the economic evaluation was included in the trial protocol paper [1]. Reporting of this study was guided by the Consolidated Health Economic Evaluation Reporting Standards (CHEERS) 2022 checklist [13]. The CHEERS 2022 checklist for this study, completed by GAJ and NB, is included as supplementary material. All analyses were carried out in Stata 18 and Microsoft Excel.

### Provider costs

Provider costs were estimated for all participants enrolled in the trial for 2 months or longer (*n* = 6714 participants) using a combination of top-down and bottom-up approaches [14]. Key summary statistics for the provider costs sample are provided in Appendix Table S1. We estimated both financial and economic costs by capturing and assigning current market value to donated goods and volunteer time. Costs were separated into capital costs, which were annuitized at 3% [7], and recurrent costs which included personnel, medication, diagnostics and facility overheads (equipment and furniture, rental space and administration).

We used a standardised facility-based costing tool in Excel to capture facility capital, personnel and overheads costs [7]. Primary data on these costs were collected during the piloting of integrated care for 10 representative health facilities [7]. Mean costs per visit and by health condition were calculated in the tool using a top-down approach based on monthly patient numbers. Medication and diagnostics costs were estimated using a ingredients approach [15], or bottom-up approach, where volumes of medication and diagnostic units provided to trial participants were multiplied by their corresponding prices per unit provided. Appendix Table S2 contains the intervention resources included and costed for each health condition and Table S3 a detailed list of medication and diagnostic prices used, along with corresponding sources.

For participants with comorbidities receiving integrated care, we assumed facility personnel and overhead costs were equivalent to those required to care for HIV alone rather than summing across costs for each of the participant’s conditions [7,16]. This assumption is supported by trial data on the mean time spent at the facility by participants per integrated care visit (Appendix Table S4), which is similar for participants with single and multiple conditions in Tanzania (mean difference of 0.1480 hours, *p* = 0.4620). In Uganda, while the mean time spent at the facility by participants receiving integrated care was higher by 0.9940 hours (*p* < 0.0001) for multiple conditions compared with single conditions, a small share of this is likely to have been attributable solely to clinician contact time. For patients with multiple morbidities receiving standard vertical care, we defined a visit as the sum of each visit required to treat their different conditions to standardise results and facilitate comparability with mean costs per integrated care visit. In other words, for a patient living with HIV and diabetes, the cost per visit would comprise the costs of both their HIV visit and their diabetes visit.

### Patient and societal costs

Patient costs were captured using an interviewer-administered questionnaire between 5 and 9 months of follow-up, which asked participants about their visit to the health facility on that day. The questionnaire was administered consecutively to all trial participants from the start of the trial. As it was taking time to complete, we reviewed the data at an interim time point, when over 2000 participants had been enrolled. The analyses of these data showed that this number was sufficient for health economics considerations. We therefore stopped these data collection. At that time, 2708 participants had been enrolled. Key summary statistics for the patient cost sample are included in the Appendix (Table S5). Medication, other medical (e.g. diagnostic tests or consultation fees) and travel (transportation and food) expenses were self-reported by participants in local currency, along with the time they spent travelling and at the facility. Participants were also asked whether they had to arrange any childcare, and for how long, as a result of their visit on that day. All time losses, including the cost of household caregiver time, were summed and valued using mean per capita consumption data from national household budget survey reports [17,18]. Mean monthly per capita consumption data were inflated to 2021 prices in both countries [19], divided by 30 to convert to daily per capita consumption and then by eight hours based on a working day to generate mean consumption per working hour. The latter was then applied to value participant travel, facility and caregiving time. We therefore valued all participant time, regardless of employment status [20].

We modelled costs per visit for participants receiving standard care. Time spent travelling, at the facility and any household caregiver time reported by participants with multiple health conditions receiving standard vertical care for that day were multiplied by two [1], to reflect the cost of treating each of their health conditions – which needed separate clinician consultations in different clinics (i.e. HIV, diabetes or hypertension). Medication and other medical expenses incurred by participants receiving standard care were assumed to be equal to participants receiving integrated care for the same health condition at an equivalent health facility level.

Provider, patient, and societal (provider and patient) costs were reported separately for Uganda and Tanzania. Provider costs were reported for 6714 participants, while patient and societal costs were reported for the sub-sample of 2708 participants. All costs were captured in local currency amounts (Ugandan or Tanzanian Shillings, Ugx or Tsh), and inflated to 2021 prices [19]. For ease of reporting and comparison, and to better account for differences in purchasing power parity between contexts, all costs were reported in 2021 International Dollars (Int$) based on World Bank exchange rates (Int$1 = Ugx1310.6 and Tsh890.58) [19]. Two-sided t-tests were used at a 5% significance level to evaluate statistically significant differences in cost between integrated and standard vertical care arms.

### Provider costs at scale

To estimate costs at scale, we used mean monthly provider costs, which were calculated and presented by health condition, country, trial arm and cost-component in the published trial paper [8]. These mean monthly provider costs were annualised and combined with national prevalence estimates for each health condition [21–23] that we disaggregated using proportions for each health condition derived from the INTE-AFRICA provider cost sample and the health facility levels at which care was sought. Total costs at scale were estimated for existing service coverage levels [21–23], and target coverage levels of 95% for HIV [24] and 80% for diabetes and hypertension [25]. Prevalence, service coverage and proportions for each health condition used to inform costs at scale are contained in Appendix Tables S6a-6d. We evaluated budget impact by dividing the difference in cost of delivering integrated versus standard care at scale by national health expenditure [19].

### Sensitivity analysis

A key assumption made to estimate mean provider costs per visit was that facility personnel and overhead costs of integrated care for participants with multiple health conditions were equivalent to those for HIV alone. We carried out a sensitivity analysis to vary this assumption and test whether integrated care remained significantly cost-saving compared with standard care after increasing personnel and overhead costs for integrated care by + 10%, +20% and + 50%. We also carried out a two-way sensitivity analysis to investigate uncertainty around estimated costs at scale. To do this, we varied the proportions of total prevalence by health condition and mean monthly provider costs per participant within 95% confidence intervals. In addition, sensitivity analyses also investigated the impact of varying assumptions used to value time loss on patient cost results. Namely, by converting monthly consumption to daily amounts assuming 22 rather than the conservative 30 working days per month and by using median monthly salaries rather than estimates of monthly consumption.

### Ethics statement, role of the funder, patient and public involvement

The INTE-AFRICA study adhered to Declaration of Helsinki principles and received approval from the ethics committees of The AIDS Support Organisation in Uganda (ref: TASOREC/090/19-UG-REC-009), National Institute of Medical Research in Tanzania (ref: NIMR/HQ/8.a/Vol. IX/3394) and the Liverpool School of Tropical Medicine in the United Kingdom (ref: 19–100). All trial participants provided written informed consent. The trial was registered at the International Standard Randomised Controlled Trial Number registry (ISRCTN43896688). Funding for the trial was from the European Union’s Horizon 2020 research and innovation program (grant agreement No: 825698). The funder had no role in data collection, analysis, interpretation of the findings, writing of the paper or the decision to submit the findings for publication. Patient and public involvement in the INTE-AFRICA trial is detailed elsewhere [1,8]. Policymakers were engaged during the economic evaluation presented in this paper, to ensure that objectives aligned with key policy questions.

## Results

Table 1 presents mean provider costs per participant visit by health condition in the integrated and standard care arms for both countries. Overall, mean provider costs per visit were higher for HIV than for hypertension (Int$ 91.12, 95% CI 88.12, 94.12, *p* < 0.0001) or diabetes (Int$ 105.70, 95% CI 100.55, 110.86, *p* < 0.0001), higher for multiple conditions than for single conditions (Int$ 15.64, 95% CI 11.69, 19.59, *p* < 0.0001) and higher for Uganda than Tanzania (Int$ 40.04, 95% CI 36.81, 43.27, *p* < 0.0001). No statistical difference (Int$ −0.02, 95% CI −3.69, 3.66, *p* = 0.9926) was found between trial arms in the mean provider cost per participant visit for single health conditions. Compared with standard care for participants with multiple health conditions, integrated clinics were estimated to save providers a mean of Int$ 18.67 (95% CI 10.89, 26.45, *p* < 0.0001) per participant visit.

**Table 1.** Mean provider costs per participant visit (2021 int$).

Mean provider costs per participant visit						
	UGANDA			TANZANIA		
	Integrated care	Standard care	Mean difference in cost (95%CI); p-value*	Integrated care	Standard care	Mean difference in cost (95%CI); p-value*
	Mean (SD)	Mean (SD)		Mean (SD)	Mean (SD)	
HIV ALONE	168.85 (45.49)	173.04 (45.35)	4.19 (−0.42, 8.80); *p* = 0.0746	152.14 (56.15)	158.69 (54.24)	6.54 (1.14, 11.95); *p* = 0.0177
HTN ALONE	107.31 (38.13)	106.81 (28.08)	−0.50 (−4.93, 3.93); *p* = 0.8250	27.18 (9.82)	27.57 (8.67)	0.39 (−0.97, 1.74); *p* = 0.5754
DM ALONE	84.17 (9.30)	85.03 (15.63)	0.85 (−3.02, 4.73); *p* = 0.6648	29.82 (3.60)	32.32 (7.50)	2.50 (0.57, 4.44); *p* = 0.0114
HIV+HTN	196.60 (67.51)	249.95 (90.03)	53.35 (33.65, 73.06); *p* < 0.0001	143.75 (57.35)	166.16 (59.96)	22.41 (9.62, 35.20); *p* = 0.0006
HIV+DM	195.35 (57.93)	246.42 (59.21)	51.07 (7.26, 94.88); *p* = 0.0239	144.81 (67.93)	170.92 (74.59)	26.11 (−11.18, 63.40); *p* = 0.1664
HTN+DM	105.14 (34.22)	176.85 (38.04)	71.72 (65.10, 78.34);*p* < 0.0001	39.73 (11.07)	56.16 (11.95)	16.43 (13.69, 19.17); *p* < 0.0001
HIV+HTN+DM	188.13 (45.79)	313.50 (84.43)	125.37 (82.75, 167.99); *p* < 0.0001	128.27 (47.62)	185.43 (65.07)	57.17 (27.23, 87.10); *p* = 0.0003

Note: SD = Standard Deviation.

*Mean difference in costs, two-sided test at a 5% significance level.

As shown in Table 1, mean provider cost-savings per participant visit were statistically significant for all participants with multiple health conditions in Uganda. In Tanzania, mean provider costs per participant visit in integrated clinics were significantly lower for most participants with multiple health conditions. Mean provider costs per participant visit for participants with concurrent HIV and diabetes receiving integrated care were lower compared with standard care, but not statistically significant (Int$ 26.11, 95% CI −11.18, 63.40, *p* = 0.1664). This is likely due to large variations in costs (integrated care sd = 67.93, standard care sd = 74.59), along with a small sample size.

Significant mean provider cost-savings from integrated care, compared with standard care for participants with multiple health conditions, were driven primarily by savings in personnel and overhead costs in both countries (Appendix Figure S1). Full breakdowns of mean provider costs per visit for each country, trial arm, study site, and by cost component are included in the Appendix (Appendix Table S7 and Figures S1-S2). Mean provider cost-savings per integrated care visit, compared with standard care for participants with multiple health conditions, were robust to variations in key assumptions (Appendix Table S8). Cost-savings remained significant following increases of up to 50% in integrated care personnel and overhead costs compared with those used in the base case analysis presented here.

Mean patient costs per visit were estimated for a sub-sample of 2708 patients. Table 2 reports the mean costs per visit incurred by participants receiving care in both arms. Overall, mean patient costs per visit were lower for participants with HIV than with either hypertension (Int$ −9.61, 95% CI −11.09, −8.14, *p* < 0.0001) or diabetes (Int$ −10.38, 95% CI −12.82, −7.95, *p* < 0.0001) and higher in Uganda than in Tanzania (Int$ 6.36, 95% CI 5.00, 7.72, *p* < 0.0001). No statistical difference (Int$ 0.39, 95% CI −1.04, 1.81, *p* = 0.5924) was found between trial arms in the mean cost per visit incurred by participants receiving care for single conditions. Participants receiving integrated care for multiple health conditions saved a mean of Int$ 5.86 (95% CI 2.57, 9.16, *p* = 0.0005) per visit compared with participants receiving standard care.

**Table 2.** Mean costs per visit incurred by patients (2021 int$).

Mean patient costs per patient visit						
	UGANDA			TANZANIA		
	Integrated care	Standard care	Mean difference in cost (95%CI); p-value*	Integrated care	Standard care	Mean difference in cost (95%CI); p-value*
	Mean (SD)	Mean (SD)		Mean (SD)	Mean (SD)	
HIV ALONE	10.97 (12.57)	11.81 (15.24)	0.84 (−1.41, 3.09); *p* = 0.4627	6.38 (6.18)	7.58 (10.67)	1.21 (−0.07, 2.48); *p* = 0.0632
HTN ALONE	21.13 (25.24)	19.08 (14.37)	−2.05 (−6.14, 2.04); *p* = 0.3252	17.30 (29.68)	15.02 (12.71)	−2.28 (−8.66, 4.09); *p* = 0.4813
DM ALONE	15.79 (19.28)	26.33 (36.77)	10.54 (−3.40, 24.48); *p* = 0.1361	18.14 (12.70)	15.50 (9.56)	−2.64 (−9.08, 3.80); *p* = 0.4144
HIV+HTN	18.81 (17.90)	21.66 (15.42)	2.84 (−3.69, 9.38); *p* = 0.3903	10.47 (10.82)	17.07 (12.78)	6.60 (2.03, 11.17); *p* = 0.0051
HIV+DM	17.35 (18.65)	35.59 (44.50)	18.24 (−14.99, 51.47); *p* = 0.2602	9.92 (6.33)	17.87 (16.57)	7.95 (−4.80, 20.71); *p* = 0.2088
HTN+DM	25.90 (24.47)	34.33 (18.64)	8.43 (2.46, 14.39); *p* = 0.0058	30.50 (31.93)	27.48 (19.61)	−3.02 (−12.69, 6.65); *p* = 0.5372
HIV+HTN+DM	27.58 (18.52)	30.24 (14.46)	2.66 (−10.74, 16.05); *p* = 0.6861	17.26 (15.85)	26.39 (11.16)	9.13 (−6.39, 24.65); *p* = 0.2262

Note: SD = Standard Deviation.

*Mean difference in costs, two-sided test at a 5% significance level.

Lower mean costs per visit incurred by participants receiving integrated care for multiple conditions, compared with standard care, were mainly due to lower travel costs and lower productivity losses (Appendix Figures S3). However, there was substantial variation in both trial arms and countries in the mean costs per visit incurred by participants receiving care for multiple health conditions (Table 2). Lower mean patient costs in the integrated care arm compared with standard care were only found to be significant at a 5% level for participants with concurrent hypertension and diabetes in Uganda (Int$ 8.43, 95% CI 2.46, 14.39, *p* = 0.0058) and concurrent HIV and hypertension (Int$ 6.60 (95% CI 2.03, 11.17, *p* = 0.0051) in Tanzania.

Mean costs per visit incurred by participants in both countries and trial arms were driven primarily by medication and travel costs (Appendix Figure S3). In Tanzania, substantial variability was observed in medication costs reported by participants. We consider the implications of this variability in the discussion section below. A full breakdown of patient costs by country, trial arm and by cost component is included in the Appendix (Appendix Table S9 and Figure S3). Patient cost results were robust to variations in assumptions used to value time loss, with similar statistical significance found across sensitivity analyses for the mean difference in costs incurred by patients per integrated care visit compared with standard care (Appendix Tables S10 and S11).

Mean societal costs per visit for participants receiving integrated care, the sum of provider, patient and caregiver costs, are presented in Table 3 by country, trial arm and health condition. Overall, mean societal costs per visit were higher for HIV than for hypertension (Int$ 72.43, 95% CI 67.39, 77.47, *p* < 0.0001) or diabetes (Int$ 85.24, 95% CI 75.68, 94.80, *p* < 0.0001), higher for multiple conditions than for single conditions (Int$ 22.64, 95% CI 16.65, 28.63, *p* < 0.0001) and higher for Uganda than Tanzania (Int$ 41.92, 95% CI 37.11, 46.72, *p* < 0.0001). Integrated care resulted in significant mean societal cost-savings per visit compared with standard care for both single (Int$ 6.56, 95% CI 1.20, 11.93, *p* = 0.0165) and multiple health conditions (Int$ 41.54, 95% CI 29.42, 53.67, *p* < 0.0001). Integrated care was significantly cost-saving compared with standard care from a societal perspective for all four multiple health conditions in Uganda and two of the four multiple health conditions in Tanzania (Table 3). When disaggregated by country and health condition, societal costs per visit for integrated care were comparable with standard care for participants with single conditions in both countries. Societal costs were primarily determined by provider costs, which accounted for 89.02% (sd = 9.49) of societal costs in Uganda and 86.48% (sd = 16.08) in Tanzania.

**Table 3.** Mean societal costs per participant visit (2021 int$).

	UGANDA (2021 Int$)			TANZANIA (2021 Int$)		
	Integrated	Standard	Difference	Integrated	Standard	Difference
Health condition	Mean (SD)	Mean (SD)	Mean difference in cost (95%CI); p-value*	Mean (SD)	Mean (SD)	Mean difference in cost (95%CI); p-value*
HIV ALONE	177.52 (40.27)	185.30 (50.31)	7.78 (0.42, 15.14); *p* = 0.0384	150.32 (54.82)	154.27 (54.77)	3.95 (−3.88, 11.79); *p* = 0.3220
HTN ALONE	124.39 (33.78)	120.70 (28.18)	−3.69 (−9.99, 2.60); *p* = 0.2489	40.92 (30.18)	39.64 (14.47)	−1.28 (−7.88, 5.33); *p* = 0.7037
DM ALONE	97.66 (22.71)	111.00 (42.84)	13.34 (−2.93, 29.61); *p* = 0.1065	45.92 (12.69)	44.77 (10.11)	−1.15 (−7.80, 5.50); *p* = 0.729
HIV+HTN	197.50 (41.39)	253.72 (80.73)	56.22 (31.21, 81.22); *p* < 0.0001	143.27 (53.98)	159.51 (51.69)	16.25 (−4.25, 36.75); *p* = 0.1191
HIV+DM	198.84 (63.45)	299.31 (110.47)	100.47 (10.74, 190.20); *p* = 0.0306	178.83 (67.14)	178.69 (64.57)	−0.14 (−59.71, 59.44); *p* = 0.9963
HTN+DM	121.50 (30.11)	205.16 (39.69)	83.66 (73.95, 93.36); *p* < 0.0001	68.24 (33.68)	80.02 (19.25)	11.78 (1.91, 21.66); *p* = 0.0198
HIV+HTN+DM	214.37 (38.57)	336.72 (74.37)	122.35 (69.63, 175.07); *p* = 0.0001	122.45 (40.76)	187.59 (73.48)	65.14 (0.07, 130.20); *p* = 0.0498

Note: SD = Standard Deviation.

*Mean difference in costs, two-sided test at a 5% significance level.

Integrated care was estimated to be cost-saving at scale compared with standard care in all sensitivity analyses. The total estimated savings to providers from implementing integrated rather than standard vertical care at scale for all seven health conditions in Uganda was Int$228 million at existing coverage levels (Table 4). These savings would be equivalent to 5.3% of 2021 national health expenditure, or 0.20% of gross domestic product (GDP). In Tanzania, we estimated total savings of Int$72 million at existing coverage levels. These savings are equivalent to 1.07% of 2021 national health expenditure, or 0.04% of GDP. Appendix Tables S12 and S13 include detailed estimates and results from sensitivity analyses by health condition for both countries.

**Table 4.** Total provider costs at existing levels of service coverage (2021 int$).

	UGANDA (Current coverage)			TANZANIA (Current coverage)		
	Integrated care	Standard care	Difference (Integrated – Standard)	Integrated care	Standard care	Difference (Integrated – Standard)
	Total cost	Total cost	Difference	Total cost	Total cost	Difference
HIV alone	668,995,477	698,801,557	−29,806,079	744,234,202	738,480,466	5,753,736
HTN alone	347,416,601	354,755,964	−7,339,363	140,432,057	124,044,720	16,387,337
DM alone	61,292,465	51,949,839	9,342,627	169,257,387	143,454,269	25,803,119
HIV+HTN	139,322,728	207,122,763	−67,800,034	157,440,559	183,461,692	−26,021,133
HIV+DM	17,929,185	22,435,573	−4,506,389	30,088,514	34,742,322	−4,653,808
HTN+DM	148,242,221	258,103,172	−109,860,952	275,251,488	355,161,469	−79,909,981
HIV+HTN+DM	28,812,121	47,366,642	−18,554,521	33,546,797	42,952,128	−9,405,330
**Total**	**1,412,010,798**	**1,640,535,510**	**-228,524,712**	**1,550,251,004**	**1,622,297,064**	**-72046,061**
**% of GDP**	**1.20%**	**1.40%**	**-0.20%**	**0.89%**	**0.93%**	**-0.04%**
**% of CHE**	**32.60%**	**37.90%**	**-5.30%**	**23.13%**	**24.20%**	**-1.07%**

Note: GDP = Gross Domestic Product. CHE = Current Health Expenditure.

## Discussion

This study has demonstrated that societal costs (i.e. provider and patient costs combined) were significantly lower with integrated primary management of HIV, diabetes and hypertension than with the standard vertical care for these conditions and that scaling up integrated care services could save tens of millions of dollars each year in Tanzania and Uganda. This complements our earlier findings that integrated and standard care both achieved very high levels of retention in care and integrated care did not adversely affect rates of HIV viral suppression.

In our study, health services organised integrated care clinics as standalone services in their health facilities with relatively small numbers of participants, managed in close to real-life conditions. It is possible that when integrated care services are rolled out to all patients attending a health facility, that the savings per patient are greater through the treatment of large numbers of patients. In our study, cost-savings were driven primarily by integrated care for people with multiple conditions, through reduced duplication of services. About 23.62% (sd = 42.48) of the study population had multiple conditions. Data on the prevalence of multiple conditions in sub-Saharan Africa are lacking but the burden of disease caused by non-communicable conditions is still growing and so overall savings from integrated care will increase over time [3]. Crucially, cost-savings from the provider perspective did not occur at the expense of patient costs, which were also lower in integrated care than standard care for people with multiple conditions.

Provider costs were much higher for HIV than for diabetes or hypertension. Our estimates for the mean provider costs of HIV are in line with previous studies in sub-Saharan Africa, including in Uganda and in Tanzania [26–29]. Fewer published estimates are available on the provider costs of diabetes and hypertension care. Nonetheless, our results are within the range of mean diabetes care costs identified by a systematic review focused on the Africa region [30]. Similarly, our provider cost estimates for hypertension care are within the ranges identified by a recent systematic review focused on sub-Saharan Africa [31].

As expected, cost-savings to providers from providing integrated versus standard care for participants with multiple morbidities were driven primarily by personnel and overhead costs in both countries. This is because all participants receiving integrated care shared the same waiting areas, facility clinicians, lab technicians, pharmacies and management of medical records [8]. In turn, participants with more than one health condition who received standard care used larger facility surface areas (and corresponding overheads) and saw several clinical and lab personnel.

Costs incurred by participants were equally high in both countries and trial arms, and were particularly high among participants with diabetes or hypertension – higher than for people living with HIV. Along with travel costs, medication costs accounted for a substantial share of patient costs in both countries and varied greatly in Tanzania. As in other sub-Saharan African contexts, antiretroviral therapy is provided for free and supply is reliable, but shortages of medicines for non-communicable conditions are common [32–34]. Thus, participants in both trial arms may have had to buy these medications at the private sector during shortfalls in supply [35] – which would have inflated reported costs. It is evident that if medications supply for non-communicable conditions was reliable and medications are provided for free, as is the case for HIV, then costs incurred by patients receiving diabetes or hypertension care would be substantially lower.

In addition to mean costs and value for money, estimating total provider costs if integrated care is scaled-up nationally is key to inform policymakers, since health interventions that are cost-effective might not be affordable for governments to implement at a national scale. We showed that implementing integrated care at existing coverage levels (i.e. the existing percentage of people in need who are able to access HIV, diabetes and/or hypertension services), could save providers Int$ 229 million in Uganda and Int$ 72 million in Tanzania, compared with standard care. Given that integrated care primarily involves operational changes to service delivery and is neither contingent on additional health workers nor additional health infrastructure, cost-savings from integrated care would start to accrue in the short term for reinvestment in the health system.

Like many other countries, Tanzania and Uganda are working to define universal health coverage health benefits packages and expand their national health insurance schemes – through the Universal Health Coverage Bill in Tanzania and the National Health Insurance Scheme Bill in Uganda [36,37]. However, both governments have limited resources for health at their disposal and will not be able to include all services for which there is demand when defining their packages. In addition, resources for health will be further limited by recent cuts in overseas aid by governments in the United States, the United Kingdom and some European countries [38,39]. The estimated cost-savings from implementing integrated care at scale would help mitigate cuts in overseas aid and provide several options for improving population health and progress towards universal health coverage. For example, cost-savings could be used to increase existing service coverage for diabetes and hypertension, improve quality of care, or ensure subsidised, consistent and sufficient supplies of medications for the management of diabetes and hypertension, so that patients are not having to purchase them.

Some limitations should be considered when interpreting results presented in this paper. Our study lacked a secondary measure of general health-related quality of life, such as quality-adjusted life years (QALYs) or disability-adjusted life years (DALYs) [40]. We were therefore unable to calculate a corresponding cost per QALY or DALY that would have enabled the comparison of standardised outcomes across health conditions, interventions and country-cost-effectiveness thresholds. However, this is somewhat mitigated by findings that integrated care was cost-saving compared with standard care. Second, our study was conducted during the COVID-19 pandemic, which impacted health service provision and the number of visits made by participants. For example, in Uganda, where there were limitations on travel between districts, if the clinic was in a different district to the one that participants lived in then they would not have been able to attend their visits. That said, COVID-19 likely affected service provision in both vertical and integrated care arms. Third, to minimise participant disruption, questionnaires used to capture patient costs were administered to some participants during their visit, before all the time they spent at the facility had passed and before they had incurred all costs associated with that visit. It is unclear exactly how this may have affected patient cost results, although it likely occurred equally in both arms and is more likely to have resulted in an underestimate of patient cost-savings for integrated care. The cost-savings presented in this paper are therefore likely to be conservative estimates.

## Conclusion

Sub-Saharan African countries have made huge strides in treating HIV, but effective control of diabetes and hypertension has remained elusive, largely because of a lack of resources. Our study shows that integrated management would represent an efficient, cost-saving model of care for the management of diabetes and hypertension as well as HIV.

## Acknowledgments

Conceptualisation: GAJ, JS, NB, GM, OU, MNS, SJ, SM, MJN; Data curation: EvW; Formal analysis: GAJ; Funding acquisition: SJ, SM, MJN; Investigation: SK, JB, IN, SO; Methodology: GAJ, NB, JS; Project administration: SK, JB, JO, KR, IN, SO, AG; Supervision: NB, JS, SJ; Validation: GAJ, AG, EvW, SJ, NB; Visualisation: GAJ; Writing – original draft: GAJ; Writing – reviewing and editing: GAJ, NB, SJ, JS, SK, JB, JO, KR, IN, SO, AG, EvW, JVL, GM, OU, MNS, NKS, SM, MJN.

## Supplementary Material

INTEAFRICA EE_CHEERS.docx

Additional_RESPONDAFRICA_author_list.docx

INTEAFRICA_EE_Appendix_DE.docx

## Data Availability

Individual participant data that underlie the results reported in this article are available, after deidentification, to researchers who provide a methodologically sound proposal. Proposals should be directed to s.jaffar@ucl.ac.uk. Data will be available for 5 years. The study protocol, statistical analysis plan, and analytic code are available upon request. Source code for software used in the trial is publicly available at https://github.com/inte-africa-trial/inte-edc

## References

[1] Mfinanga SG, Nyirenda MJ, Mutungi G, et al. Integrating HIV, diabetes and hypertension services in Africa: study protocol for a cluster randomised trial in Tanzania and Uganda. BMJ Open. 2021;11:e047979. doi: 10.1136/bmjopen-2020-047979 PMC8515479 34645657

[2] Skordis-Worrall J, Round J, Arnold M, et al. Addressing the double-burden of diabetes and tuberculosis: lessons from Kyrgyzstan. Global Health. 2017;13:1–11. doi: 10.1186/s12992-017-0239-3 28298226 PMC5353796

[3] Gouda HN, Charlson F, Sorsdahl K, et al. Burden of non-communicable diseases in sub-Saharan Africa, 1990–2017: results from the global burden of disease study 2017. Lancet Global Health. 2019;7:e1375–e1387. doi: 10.1016/S2214-109X(19)30374-2 31537368

[4] Global Health Expenditure Database [Internet]. World Health Organisation; 2023 [cited 2023 Oct 3: Available from: http://apps.who.int/nha/database/ViewData/Indicators/en

[5] Chang AY, Cowling K, Micah AE, et al. Past, present, and future of global health financing: a review of development assistance, government, out-of-pocket, and other private spending on health for 195 countries, 1995–2050. Lancet. 2019;393:2233–2260. doi: 10.1016/S0140-6736(19)30841-4 31030984 PMC6548764

[6] Dieleman JL, Hanlon M. Measuring the displacement and replacement of government health expenditure. Health Econ. 2014;23:129–140. doi: 10.1002/hec.3016 24327240 PMC4229065

[7] Shiri T, Birungi J, Garrib AV, et al. Patient and health provider costs of integrated HIV, diabetes and hypertension ambulatory health services in low-income settings—an empirical socio-economic cohort study in Tanzania and Uganda. BMC Med. 2021;19:1–15. doi: 10.1186/s12916-021-02094-2 34503496 PMC8431904

[8] Kivuyo S, Birungi J, Okebe J, et al. Integrated management of HIV, diabetes, and hypertension in sub-Saharan Africa (INTE-AFRICA): a pragmatic cluster-randomised, controlled trial. Lancet. 2023;402:1241–1250. doi: 10.1016/S0140-6736(23)01573-8 37805215

[9] McCombe G, Lim J, Van Hout MC, et al. Integrating care for diabetes and hypertension with HIV care in Sub-Saharan Africa: a scoping review. Int J Integr Care. 2022;22:6. doi: 10.5334/ijic.5839 PMC8815447 35136387

[10] Turner HC, Archer RA, Downey LE, et al. An introduction to the main types of economic evaluations used for informing priority setting and resource allocation in healthcare: key features, uses, and limitations. Front Public Health. 2021;9:722927. doi: 10.3389/fpubh.2021.722927 34513790 PMC8424074

[11] Drummond MF, Sculpher MJ, Claxton K, et al. Methods for the economic evaluation of health care programmes. Oxford (UK): Oxford university press; 2015.

[12] Wilkinson T, Sculpher MJ, Claxton K, et al. The international decision support initiative reference case for economic evaluation: an aid to thought. Value in Health. 2016;19:921–928. doi: 10.1016/j.jval.2016.04.015 27987641

[13] Husereau D, Drummond M, Augustovski F, et al. Consolidated health economic evaluation reporting standards (CHEERS) 2022 explanation and elaboration: a report of the ISPOR CHEERS II good practices task force. Value in Health. 2022;25:10–31. doi: 10.1016/j.jval.2021.10.008 35031088

[14] Vassall A, Sweeney S, Kahn J, et al. Reference case for estimating the costs of global health services and interventions. 2017.

[15] Drummond M, Sculpher M. Common methodological flaws in economic evaluations. Med Care. 2005;43:II–5. doi: 10.1097/01.mlr.0000170001.10393.b7 16056003

[16] Shade SB, Osmand T, Kwarisiima D, Brown, LB, Luo, A, Mwebaza, B, Mwesigye, AR, Kwizera, E, Imukeka, H, Mwanga, F, Ayieko, J. Costs of integrating hypertension care into HIV care in rural East African clinics. AIDS. 2021;35:911. doi: 10.1097/QAD.0000000000002834 33821821 PMC8030915

[17] Uganda Bureau of Statistics. Uganda national household survey 2016/17 report. Kampala, Uganda; 2018.

[18] Tanzanian National Bureau of Statistics. Household budget survey 2017–18 - Tanzania Mainland: final report. Dodoma, Tanzania; 2020.

[19] World Bank. World bank open data: world Bank. 2023 [2023 Jan 3]. Available from: https://data.worldbank.org/

[20] Krol M, Brouwer W, Rutten F. Productivity costs in economic evaluations: past, present, future. Pharmacoeconomics. 2013;31:537–549. doi: 10.1007/s40273-013-0056-3 23620213

[21] UNAIDS. AIDSinfo: global data on HIV epidemiology and response. UNAIDS; 2023 [cited 2023 Aug]. Available from: https://aidsinfo.unaids.org/

[22] Zhou B, Carrillo-Larco RM, Danaei G, et al. Worldwide trends in hypertension prevalence and progress in treatment and control from 1990 to 2019: a pooled analysis of 1201 population-representative studies with 104 million participants. Lancet. 2021;398:957–980. doi: 10.1016/S0140-6736(21)01330-1 34450083 PMC8446938

[23] Internation Diabetes Foundation. Idf Diabetes Atlas. 10th ed. Brussels (Belgium): IDF; 2021.

[24] United Nations General Assembly. Editor political declaration on HIV and AIDS: ending inequalities and getting on track to end AIDS by 2030 2021. 2021.

[25] Watkins DA, Qi J, Kawakatsu Y, et al. Resource requirements for essential universal health coverage: a modelling study based on findings from disease control priorities. Lancet Global Health. 2020;8:e829–e839. doi: 10.1016/S2214-109X(20)30121-2 32446348 PMC7248571

[26] Jaffar S, Amuron B, Foster S, et al. Rates of virological failure in patients treated in a home-based versus a facility-based HIV-care model in Jinja, southeast Uganda: a cluster-randomised equivalence trial. Lancet. 2009;374:2080–2089. doi: 10.1016/S0140-6736(09)61674-3 19939445 PMC2806484

[27] Babigumira JB, Castelnuovo B, Stergachis A, et al. Cost effectiveness of a pharmacy-only refill program in a large urban HIV/AIDS clinic in Uganda. PLoS One. 2011;6:e18193. doi: 10.1371/journal.pone.0018193 21464895 PMC3065481

[28] Menzies NA, Berruti AA, Berzon R, et al. The cost of providing comprehensive HIV treatment in PEPFAR-supported programs. AIDS. 2011;25:1753. doi: 10.1097/QAD.0b013e3283463eec 21412127 PMC3225224

[29] Kimaro GD, Mfinanga S, Simms V, et al. The costs of providing antiretroviral therapy services to HIV-infected individuals presenting with advanced HIV disease at public health centres in Dar es Salaam, Tanzania: findings from a randomised trial evaluating different health care strategies. PLoS One. 2017;12:e0171917. doi: 10.1371/journal.pone.0171917 28234969 PMC5325220

[30] Mutyambizi C, Pavlova M, Chola L, et al. Cost of diabetes mellitus in Africa: a systematic review of existing literature. Global Health. 2018;14:1–13. doi: 10.1186/s12992-017-0318-5 29338746 PMC5771003

[31] Gnugesser E, Chwila C, Brenner S, et al. The economic burden of treating uncomplicated hypertension in Sub-Saharan Africa: a systematic literature review. BMC Public Health. 2022;22:1507. doi: 10.1186/s12889-022-13877-4 35941626 PMC9358363

[32] Bintabara D, Ngajilo D. Readiness of health facilities for the outpatient management of non-communicable diseases in a low-resource setting: an example from a facility-based cross-sectional survey in Tanzania. BMJ Open. 2020;10:e040908. doi: 10.1136/bmjopen-2020-040908 PMC7661355 33177143

[33] Bintabara D, Shayo FK. Disparities in availability of services and prediction of the readiness of primary healthcare to manage diabetes in Tanzania. Prim Care Diabetes. 2021;15:365–371. doi: 10.1016/j.pcd.2020.11.007 33262058

[34] Katende D, Mutungi G, Baisley K, et al. Readiness of Ugandan health services for the management of outpatients with chronic diseases. Trop Med Int Health. 2015;20:1385–1395. doi: 10.1111/tmi.12560 26095069 PMC4758403

[35] Shannon GD, Haghparast-Bidgoli H, Chelagat W, et al. Innovating to increase access to diabetes care in Kenya: an evaluation of Novo Nordisk’s base of the pyramid project. Glob Health Action. 2019;12:1605704. doi: 10.1080/16549716.2019.1605704 31116677 PMC6534215

[36] Namyalo PK, Mutatina B, Byakika S, et al. The feasibility analysis of integrating community-based health insurance schemes into the national health insurance scheme in Uganda. PLoS One. 2023;18:e0284246. doi: 10.1371/journal.pone.0284246 37058490 PMC10104299

[37] Mori AT. Mandatory health insurance for the informal sector in Tanzania—has it worked anywhere! Front Health Serv. 2023;3:1247301. doi: 10.3389/frhs.2023.1247301 37849823 PMC10577424

[38] Kyobutungi C, Okereke E, Abimbola S. After USAID: what now for aid and Africa? BMJ. 2025;388:r479. doi: 10.1136/BMJ.r479 40068845

[39] Olivia O’Sullivan JP. First USAID closes, then UK cuts aid: what a western retreat from foreign aid could mean. Chatham House; 2025. Available from: https://www.chathamhouse.org/2025/03/first-usaid-closes-then-uk-cuts-aid-what-western-retreat-foreign-aid-could-mean

[40] Herdman M, Gudex C, Lloyd A, et al. Development and preliminary testing of the new five-level version of EQ-5D (EQ-5D-5L). Qual Life Res. 2011;20:1727–1736. doi: 10.1007/s11136-011-9903-x 21479777 PMC3220807

