# Supplementary material for: Estimated cost-savings from integrated care for HIV, diabetes and hypertension in sub-Saharan Africa: a cost-minimisation analysis
Source: Glob Health Action. 2025 Sep 9;18(1):2556364. doi: 10.1080/16549716.2025.2556364 (PMC12422034; doi:10.1080/16549716.2025.2556364)
Supplement: INTEAFRICA EE_CHEERS.docx [file ZGHA_A_2556364_SM4709.docx]

## Consolidated Health Economic Evaluation Reporting Standards (CHEERS) 2022 checklist

| **Topic** | **No.** | **Item** | **Location where item is reported** |
| --- | --- | --- | --- |
| **Title** |  |  |  |
|  | 1 | Identify the study as an economic evaluation and specify the interventions being compared. | Title |
| **Abstract** |  |  |  |
|  | 2 | Provide a structured summary that highlights context, key methods, results, and alternative analyses. | Abstract |
| **Introduction** |  |  |  |
| **Background and objectives** | 3 | Give the context for the study, the study question, and its practical relevance for decision making in policy or practice. | “Research in Context” and “Introduction” |
| **Methods** |  |  |  |
| **Health economic analysis plan** | 4 | Indicate whether a health economic analysis plan was developed and where available. | “Economic evaluation objective” in “Methods”. Referred to the main trial protocol for the health economic analysis plan. |
| **Study population** | 5 | Describe characteristics of the study population (such as age range, demographics, socioeconomic, or clinical characteristics). | Tables S1 and S5 in Appendix. |
| **Setting and location** | 6 | Provide relevant contextual information that may influence findings. | “INTE-AFRICA trial” and “Economic evaluation objective” in “Methods” and paragraphs 3 and 5 in “Discussion”. |
| **Comparators** | 7 | Describe the interventions or strategies being compared and why chosen. | “Vertical and integrated care” in “Methods” |
| **Perspective** | 8 | State the perspective(s) adopted by the study and why chosen. | “Economic evaluation objective” in “Methods” |
| **Time horizon** | 9 | State the time horizon for the study and why appropriate. | “Economic evaluation objective” in “Methods” |
| **Discount rate** | 10 | Report the discount rate(s) and reason chosen. | Not applicable (12-month time-horizon) |
| **Selection of outcomes** | 11 | Describe what outcomes were used as the measure(s) of benefit(s) and harm(s). | “INTE-AFRICA trial” and “Economic evaluation objective” in “Methods” |
| **Measurement of outcomes** | 12 | Describe how outcomes used to capture benefit(s) and harm(s) were measured. | “INTE-AFRICA trial” in “Methods” |
| **Valuation of outcomes** | 13 | Describe the population and methods used to measure and value outcomes. | Not applicable |
| **Measurement and valuation of resources and costs** | 14 | Describe how costs were valued. | “Provider costs” and “Patient and societal costs” in “Methods” and Tables S2 and S3 in Appendix. |
| **Currency, price date, and conversion** | 15 | Report the dates of the estimated resource quantities and unit costs, plus the currency and year of conversion. | “Provider costs” and “Patient and societal costs” in “Methods” and Tables S2 and S3 in Appendix. |
| **Rationale and description of model** | 16 | If modelling is used, describe in detail and why used. Report if the model is publicly available and where it can be accessed. | Not applicable |
| **Analytics and assumptions** | 17 | Describe any methods for analysing or statistically transforming data, any extrapolation methods, and approaches for validating any model used. | “Provider costs”, “Patient and societal costs”, “Provider costs at scale” and “Sensitivity analysis” in “Methods” |
| **Characterising heterogeneity** | 18 | Describe any methods used for estimating how the results of the study vary for subgroups. | “Provider costs” and “Patient and societal costs” in “Methods” and Tables S2 and S3 in Appendix. |
| **Characterising distributional effects** | 19 | Describe how impacts are distributed across different individuals or adjustments made to reflect priority populations. | Not applicable |
| **Characterising uncertainty** | 20 | Describe methods to characterise any sources of uncertainty in the analysis. | “Sensitivity analysis” in “Methods”. |
| **Approach to engagement with patients and others affected by the study** | 21 | Describe any approaches to engage patients or service recipients, the general public, communities, or stakeholders (such as clinicians or payers) in the design of the study. | Not applicable |
| **Results** |  |  |  |
| **Study parameters** | 22 | Report all analytic inputs (such as values, ranges, references) including uncertainty or distributional assumptions. | Tables S2, S3 and S6a-S6d in Appendix. |
| **Summary of main results** | 23 | Report the mean values for the main categories of costs and outcomes of interest and summarise them in the most appropriate overall measure. | “Results” text, Tables 1-4 in “Results”, Tables S7 and S9 and Figures S1 and S3 in Appendix |
| **Effect of uncertainty** | 24 | Describe how uncertainty about analytic judgments, inputs, or projections affect findings. Report the effect of choice of discount rate and time horizon, if applicable. | Paragraph 3 in “Results”, Tables S8 and S10-12 in Appendix. |
| **Effect of engagement with patients and others affected by the study** | 25 | Report on any difference patient/service recipient, general public, community, or stakeholder involvement made to the approach or findings of the study | Not applicable |
| **Discussion** |  |  |  |
| **Study findings, limitations, generalisability, and current knowledge** | 26 | Report key findings, limitations, ethical or equity considerations not captured, and how these could affect patients, policy, or practice. | “Discussion” |
| **Other relevant information** |  |  |  |
| **Source of funding** | 27 | Describe how the study was funded and any role of the funder in the identification, design, conduct, and reporting of the analysis | “Ethics statement, role of the funder, patient and public involvement” in “Methods” |
| **Conflicts of interest** | 28 | Report authors conflicts of interest according to journal or International Committee of Medical Journal Editors requirements. | “Conflict of interest” |
