# Supplementary material for: Estimated cost-savings from integrated care for HIV, diabetes and hypertension in sub-Saharan Africa: a cost-minimisation analysis
Source: Glob Health Action. 2025 Sep 9;18(1):2556364. doi: 10.1080/16549716.2025.2556364 (PMC12422034; doi:10.1080/16549716.2025.2556364)
Supplement: Additional_RESPONDAFRICA_author_list.docx [file ZGHA_A_2556364_SM4708.docx]

Others members of RESPOND AFRICA:

| **First name** | **Last name** | **Highest degree** | **E-mail** |
| --- | --- | --- | --- |
| Prof Duolao | Wang | PhD | duolao.wang@lstmed.ac.uk |
| Samafilan | Ainan | MMed | samafilan@gmail.com |
| Joshua | Musinguzi | MPH | joshuamusinguzi@yahoo.co.uk |
| Caroline | Jeffery | PhD | c.jeffery@liverpool.ac.uk |
| Prof Geoff | Gill | PhD | g.gill@liverpool.ac.uk |
| Prof Peter G | Smith | Dsc | peter.smith@lshtm.ac.uk |
| Prof Anne R | Katahoire | PhD | anne.ruhweza.katahoire@gmail.com |
| Prof Max | Bachmann | PhD | m.bachmann@uea.ac.uk |
| Faith | Moyo | PhD | f.moyo@ucl.ac.uk |
| Katie | Bates | PhD | katie.bates@ucl.ac.uk |
| Prof Marie-Claire | van Hout | PhD | m.c.vanhout@ljmu.ac.uk |
| Elizabeth | Shayo | PhD | bethshayo73@gmail.com |
