# Supplementary material for: Estimated cost-savings from integrated care for HIV, diabetes and hypertension in sub-Saharan Africa: a cost-minimisation analysis
Source: Glob Health Action. 2025 Sep 9;18(1):2556364. doi: 10.1080/16549716.2025.2556364 (PMC12422034; doi:10.1080/16549716.2025.2556364)
Supplement: INTEAFRICA_EE_Appendix_DE.docx [file ZGHA_A_2556364_SM4707.docx]

**Appendix file 1 for: “Estimated cost-savings from integrated care for HIV, diabetes and hypertension in sub-Saharan Africa: A cost-minimisation analysis”**

[Table S1. Provider cost sample characteristics, number of participants (percent). 2](#_Toc207440883)

[Table S2. Resource use by condition(1) 4](#_Toc207440884)

[Table S3. Diagnostic and medication prices in 2021 local currency units 5](#_Toc207440885)

[Table S4. Mean time spent at the facility by participants per visit and time spent travelling to the facility 8](#_Toc207440886)

[Table S5. Patient cost sub-sample characteristics, number of participants (percent). 10](#_Toc207440887)

[Table S6a. Population size by country 13](#_Toc207440888)

[Table S6b. Number of people living with HIV, hypertension and diabetes by country 13](#_Toc207440889)

[Table S6c. Proportion of each health condition from INTE-AFRICA trial data with 95%CIs 13](#_Toc207440890)

[Table S6d. Existing and target service coverage levels by country 14](#_Toc207440891)

[Table S7. Mean provider costs per patient visit by cost component in Uganda and Tanzania (2021 Int$) 16](#_Toc207440892)

[Table S8. Personnel and overheads sensitivity analyses for mean costs per patient visit (2021 Int$) 20](#_Toc207440893)

[Table S9. Mean patient costs per patient visit by cost component in Uganda and Tanzania (2021 Int$) 22](#_Toc207440894)

[Table S10. Sensitivity analysis for patient costs per visit, assuming 22 working days per month to value time loss (2021 Int$) 27](#_Toc207440895)

[Table S11. Sensitivity analysis for patient costs per visit, using salaries to value time loss (2021 Int$) 27](#_Toc207440896)

[Table S12. Sensitivity analyses for costs at scale (2021 Int$) 29](#_Toc207440897)

[Table S13. Total provider costs at target levels of service coverage (2021 Int$) 32](#_Toc207440898)

[Figure S1. Mean provider costs per visit for patients receiving integrated or standard care, by cost component 33](#_Toc207440899)

[Figure S2. Mean provider costs per patient visit by health facility in Uganda and Tanzania (2021 Int$) 34](#_Toc207440900)

[Figure S3. Mean patient costs per visit for patients receiving integrated or vertical care, by cost component 36](#_Toc207440901)

##

## Table S1. Provider cost sample characteristics, number of participants (percent).

|  | **Uganda** | | **Tanzania** | |
| --- | --- | --- | --- | --- |
|  | **Integrated care**  **(n=1738)** | **Standard care**  **(n=** **1650)** | **Integrated care**  **(n=** **1535)** | **Standard care**  **(n=** **1791)** |
| ***Health condition*** | | | | |
| HIV alone | 717 | 782 | 815 | 790 |
|  | 41.25% | 47.39% | 53.09% | 44.11% |
| HTN alone | 490 | 417 | 341 | 379 |
|  | 28.19% | 25.27% | 22.21% | 21.16% |
| DM alone | 79 | 113 | 65 | 140 |
|  | 4.55% | 6.85% | 4.23% | 7.82% |
| HIV+HTN | 174 | 85 | 163 | 163 |
|  | 10.01% | 5.15% | 10.62% | 9.10% |
| HIV+DM | 15 | 15 | 25 | 37 |
|  | 0.86% | 0.91% | 1.63% | 2.07% |
| HTN+DM | 242 | 218 | 99 | 248 |
|  | 13.92% | 13.21% | 6.45% | 13.85% |
| HIV+HTN+DM | 21 | 20 | 27 | 34 |
|  | 1.21% | 1.21% | 1.76% | 1.90% |
| ***Sex*** | | | | |
| Female | 1334 | 1193 | 1113 | 1286 |
|  | (76.75%) | (72.30%) | (72.51%) | (71.80%) |
| Male | 404 | 457 | 422 | 505 |
|  | (23.25%) | (27.70%) | (27.49%) | (28.20%) |
| ***Education level*** | | | | |
| No formal education | 289 | 200 | 270 | 205 |
|  | (19.82%) | (13.84%) | (19.33%) | (12.80%) |
| Primary school | 841 | 955 | 907 | 1098 |
|  | (57.68%) | (66.09%) | (64.92%) | (68.54%) |
| Secondary school or higher | 328 | 290 | 220 | 299 |
|  | (22.50%) | (20.07%) | (15.75%) | (18.66%) |
| ***Age in years*** | | | | |
| <35 | 322 | 281 | 163 | 177 |
|  | (18.53%) | (17.03%) | (10.62%) | (9.88%) |
| 35–49 | 567 | 595 | 576 | 665 |
|  | (32.62%) | (36.06%) | (37.52%) | (37.13%) |
| >=50 | 849 | 774 | 796 | 949 |
|  | (48.85%) | (46.91%) | (51.86%) | (52.99%) |
| ***BMI, kg/m2*** | | | | |
| <25 | 980 | 966 | 665 | 787 |
|  | (56.39%) | (58.55%) | (43.35%) | (43.94%) |
| 25-29 | 457 | 438 | 463 | 539 |
|  | (26.29%) | (26.55%) | (30.18%) | (30.09%) |
| >=30 | 301 | 246 | 406 | 465 |
|  | (17.32%) | (14.91%) | (26.47%) | (25.96%) |
| ***Blood pressure, mm Hg*** | | | | |
| <140/90 | 1147 | 1042 | 892 | 976 |
|  | (66.11%) | (63.19%) | (58.15%) | (54.49%) |
| >=140 or >=90 | 588 | 607 | 642 | 815 |
|  | (33.89%) | (36.81%) | (41.85%) | (45.51%) |
| ***Fasting blood glucose 6·9 mmol/L among participants with diabetes*** | | | | |
| <7 | 87 | 80 | 36 | 71 |
|  | (32.95%) | (25.16%) | (22.78%) | (17.93%) |
| >=7 | 177 | 238 | 122 | 325 |
|  | (67.05%) | (74.84%) | (77.22%) | (82.07%) |
| ***HIV viral load, copies per mL*** | | | | |
| <1000 | 791 | 797 | 994 | 983 |
|  | (94.73%) | (92.03%) | (96.98%) | (96.75%) |
| >=1000 | 44 | 69 | 31 | 33 |
|  | (5.27%) | (7.97%) | (3.02%) | (3.25%) |
| ***HIV viral load <400 copies per mL*** | | | | |
| <400 | 690 | 669 | 985 | 966 |
|  | (82.63%) | (77.25%) | (96.10%) | (95.08%) |
| >=400 | 145 | 197 | 40 | 50 |
|  | (17.37%) | (22.75%) | (3.90%) | (4.92%) |
| ***Marital status*** | | | | |
| Divorced | 353 | 347 | 239 | 314 |
|  | (20.31%) | (21.03%) | (15.57%) | (17.53%) |
| Married | 857 | 882 | 761 | 955 |
|  | (49.31%) | (53.45%) | (49.58%) | (53.32%) |
| Single | 130 | 124 | 222 | 144 |
|  | (7.48%) | (7.52%) | (14.46%) | (8.04%) |
| Widowed | 398 | 297 | 313 | 378 |
|  | (22.90%) | (18.00%) | (20.39%) | (21.11%) |

## Table S2. Resource use by condition(1)

|  | **Single conditions** | | | **Multiple conditions** | | | |
| --- | --- | --- | --- | --- | --- | --- | --- |
| **Cost component** | **Diabetes (DM)** | **Hypertension (HTN)** | **HIV** | **DM + HTN** | **DM + HIV** | **HTN + HIV** | **DM + HTN + HIV** |
| **Staff** | Triage, Nurse, Clinician, Pharmacist, Laboratory technician | | | | | | |
|  |  |  |  |  |  |  |  |
| **Medication** | Diabetes drugs | BP drugs | Antiretroviral drugs | Diabetes drugs | Diabetes drugs | BP drugs | Diabetes drugs |
|  |  |  |  | BP drugs | ART + HIV-related complications | ART + HIV-related complications | BP drugs |
|  |  |  |  |  |  |  | ART + HIV-related complications |
|  |  |  |  |  |  |  |  |
| **Laboratory** | RBS, FBS, LFT, RFT | LFT, RFT, Lipids, RBS, | CD4 count, VL, FBC | RBS, FBS, LFT, RFT, | RBS, FBS, LFT, RFT, | LFT, RFT, CD4 count | RBS, FBS, LFT, RFT, Lipids, |
|  | HBA1C, Lipids, FBP | Creatinine, FBS, | Haemoglobin, RBS, | HBA1C, Lipids, BUN, | HBA1C, CD4 count, VL, | VL, Lipids | HBA1C, CD4 count, VL |
|  | Creatinine, BUN, | MRDT, Urinalysis | BUN, creatinine, | Urinalysis, creatinine | Lipids, |  |  |
|  | Urinalysis |  | HBA1C, haemoglobin |  |  |  |  |
| **Overheads** |  |  |  |  |  |  |  |
| *Administration* | NCD admin | NCD admin | CTC admin | NCD admin | NCD or CTC admin | NCD or CTC admin | NCD or CTC admin |
|  |  |  |  |  |  |  |  |
| *Rental space* | NCD space | NCD space | CTC space | NCD space | NCD or CTC space | NCD or CTC space | NCD or CTC space |
|  |  |  |  |  |  |  |  |
| *Furniture* | NCD furniture | NCD furniture | CTC furniture | NCD furniture | NCD or CTC furniture | NCD or CTC furniture | NCD or CTC furniture |
|  |  |  |  |  |  |  |  |
| *Equipment* | Glucometers | BP machines | Weighing scales | Glucometers | Glucometers | BP machines | Glucometers |
|  | Weighing scales | Weighing scales | Stethoscopes | Weighing scales | Weighing scales | Weighing scales | Weighing scales |
|  | Stethoscopes | Stethoscopes | BP machines | Stethoscopes | Stethoscopes | Stethoscopes | Stethoscopes |
|  | BP machines |  |  | BP machines | BP machines |  | BP machines |

## Table S3. Diagnostic and medication prices in 2021 local currency units

| **Resource item** | **Uganda (UGX)** | **Source** | **Tanzania (TSh)** | **Source** |
| --- | --- | --- | --- | --- |
| **Laboratory, per test** |  |  |  |  |
| Urea/Creatinine | 20440 | Sinux (Kiruddu) Laboratories | 10367 | Lancet Laboratories Tanzania |
| Urinalysis | 10220 | Sinux (Kiruddu) Laboratories | 10367 | Lancet Laboratories Tanzania |
| Full blood count | 15330 | Sinux (Kiruddu) Laboratories | 10367 | Lancet Laboratories Tanzania |
| HIV rapid testing | 10220 | Sinux (Kiruddu) Laboratories | 23740 | Lancet Laboratories Tanzania |
| CD4 count | 51100 | Sinux (Kiruddu) Laboratories | 31100 | Lancet Laboratories Tanzania |
| Viral load | 255498 | Sinux (Kiruddu) Laboratories | 124401 | Lancet Laboratories Tanzania |
| Renal function test | 40880 | Sinux (Kiruddu) Laboratories | 10367 | Lancet Laboratories Tanzania |
| Liver function (ALT) test | 40880 | Sinux (Kiruddu) Laboratories | 88014 | Lancet Laboratories Tanzania |
| Haemoglobin test | 66429 | Sinux (Kiruddu) Laboratories | 14410 | Lancet Laboratories Tanzania |
| Syphilis (VDRL) test | 10220 | Sinux (Kiruddu) Laboratories | 10367 | Lancet Laboratories Tanzania |
| Malaria RDC test | 10220 | Sinux (Kiruddu) Laboratories | 5183 | Lancet Laboratories Tanzania |
| Pregnancy test | 45990 | Sinux (Kiruddu) Laboratories | 18556 | Lancet Laboratories Tanzania |
| H. Pylori test | 15330 | Sinux (Kiruddu) Laboratories | 56913 | Lancet Laboratories Tanzania |
| Hepatitis B test | 45990 | Sinux (Kiruddu) Laboratories | 10367 | Lancet Laboratories Tanzania |
| Gene Xpert | 45990 | Sinux (Kiruddu) Laboratories | 64170 | Lancet Laboratories Tanzania |
| CrAg test | 25550 | Sinux (Kiruddu) Laboratories | 16587 | Lancet Laboratories Tanzania |
| Random blood sugar test | 10220 | Sinux (Kiruddu) Laboratories | 10263 | Lancet Laboratories Tanzania |
| Fasting blood sugar test | 10220 | Sinux (Kiruddu) Laboratories | 10263 | Lancet Laboratories Tanzania |
| HBA1C test | 35770 | Sinux (Kiruddu) Laboratories | 56913 | Lancet Laboratories Tanzania |
| OGTT test | 66429 | Sinux (Kiruddu) Laboratories | - |  |
| Lipid profile test | 40880 | Sinux (Kiruddu) Laboratories | 56913 | Lancet Laboratories Tanzania |
| **Medication** |  |  |  |  |
| ***Antiretroviral therapy per month*** |  |  |  |  |
| TDF/3TC/DTG 300mg/300mg/50mg, per 30 tablets | 33284 | National Medical Stores | 20595 | WHO Global Price Reporting Mechanism |
| AZT/3TC/NPV 300mg/150mg/200mg, per 60 tablets | 38840 | National Medical Stores | 24033 | WHO Global Price Reporting Mechanism |
| DTG 50mg, per 30 tablets | 10518 | National Medical Stores | 6508 | WHO Global Price Reporting Mechanism |
| EFV 200mg, per 30 tablets | 16792 | WHO Global Price Reporting Mechanism | 10390 | WHO Global Price Reporting Mechanism |
| RTV 100mg, per 30 tablets | 18710 | National Medical Stores | 11577 | WHO Global Price Reporting Mechanism |
| ATV/r 300mg/100mg, per 30 tablets | 61950 | WHO Global Price Reporting Mechanism | 38332 | WHO Global Price Reporting Mechanism |
| TDF/3TC/EFV 300mg/300mg/600mg, per 30 tablets | 39557 | National Medical Stores | 24477 | WHO Global Price Reporting Mechanism |
| TDF/3TC 300mg/300mg, per 30 tablets | 22766 | National Medical Stores | 14087 | WHO Global Price Reporting Mechanism |
| AZT/3TC 300mg/150mg, per 60 tablets | 40510 | National Medical Stores | 25066 | WHO Global Price Reporting Mechanism |
| LPV/r 200mg/50mg, per 120 tablets | 133788 | National Medical Stores | 82783 | WHO Global Price Reporting Mechanism |
| NVP 200mg, per 30 tablets | 23596 | WHO Global Price Reporting Mechanism | 14601 | WHO Global Price Reporting Mechanism |
| ***Diabetes treatment per month*** |  |  |  |  |
| Insulin mixtard human 100IU/ML, per 100 vials | 18254 | National Medical Stores | - |  |
| Glibenclamide 5mg, per 100 tablets | 3212 | National Medical Stores | - |  |
| Metformin HCL 500mg, per 100 tablets | 3655 | National Medical Stores | - |  |
| Glimepiride 2mg + Metformin 500mg | - |  | 9123 | Medical Stores Department |
| Glimepiride 1mg + Metformin 500mg | - |  | 6220 | Medical Stores Department |
| Losartan 50mg + Hydrochlothiazide 12.5mg | - |  | 16379 | Medical Stores Department |
| Losartan 50mg + Hydrochlothiazide 5mg | - |  | 13062 | Medical Stores Department |
| ***Hypertension treatment per month*** |  |  |  | Medical Stores Department |
| Bendroflumethiazide 5mg, per 100 tablets | 25681 | National Medical Stores | 778 | Medical Stores Department |
| Amlodipine 10mg, per 100 tablets | 7064 | National Medical Stores | 2977 | Medical Stores Department |
| Nifedipine 5mg, per 100 tablets | 7154 | National Medical Stores | 6738 | Medical Stores Department |
| Furosemide 40mg, per 1000 tablets | 11957 | National Medical Stores | 594 | Medical Stores Department |
| Enalapril 10mg, per 28 tablets | 9271 | National Medical Stores | 8501 | Medical Stores Department |
| Atenolol 100mg, per 1000 tablets | 26674 | National Medical Stores | 622 | Medical Stores Department |
| **Antibiotics** |  |  |  |  |
| Cotrimoxazole 120mg, per 100 tablets | 7767 | National Medical Stores | - |  |
| Amoxicillin/Ampicillin 250mg capsule, per 20 capsules | 542677 | National Medical Stores | - |  |
| **Other drugs** |  |  |  |  |
| Fluconazole 200mg, per 100 tablets | 206442 | National Medical Stores | - |  |

Source: Updated from (1)

## Table S4. Mean time spent at the facility by participants per visit and time spent travelling to the facility

| **UGANDA** | | | | | | | | | | |  |
| --- | --- | --- | --- | --- | --- | --- | --- | --- | --- | --- | --- |
|  | **Integrated care** | | | | | **Standard care** | | | | |  |
| **Health condition** | **Mean** | **Std. Err.** | **Std. Dev.** | **[95% Conf. Interval]** | | **Mean** | **Std. Err.** | **Std. Dev.** | **[95% Conf. Interval]** | |  |
| **HIV ALONE** |  |  |  |  |  |  |  |  |  |  |  |
| Travel time | 1.9258 | 0.1049 | 1.6053 | 1.7190 | 2.1325 | 1.7262 | 0.0570 | 1.1053 | 1.6142 | 1.8383 |  |
| Facility time | 2.1104 | 0.1316 | 2.1060 | 1.8512 | 2.3696 | 3.1425 | 0.0937 | 1.8006 | 2.9582 | 3.3268 |  |
| **HTN ALONE** |  |  |  |  |  |  |  |  |  |  |  |
| Travel time | 1.6205 | 0.1025 | 1.3127 | 1.4181 | 1.8229 | 1.7860 | 0.0964 | 1.3809 | 1.5959 | 1.9762 |  |
| Facility time | 2.9323 | 0.1918 | 2.4261 | 2.5535 | 3.3111 | 3.6594 | 0.0889 | 1.2699 | 3.4841 | 3.8347 |  |
| **DM ALONE** |  |  |  |  |  |  |  |  |  |  |  |
| Travel time | 1.6657 | 0.1583 | 0.9095 | 1.3432 | 1.9881 | 2.1870 | 0.2148 | 1.3755 | 1.7528 | 2.6211 |  |
| Facility time | 1.9436 | 0.3739 | 2.1801 | 1.1830 | 2.7043 | 3.9207 | 0.2178 | 1.3947 | 3.4805 | 4.3609 |  |
| **HIV+HTN** |  |  |  |  |  |  |  |  |  |  |  |
| Travel time | 1.8782 | 0.1700 | 1.2257 | 1.5370 | 2.2194 | 3.5490 | 0.3514 | 2.5094 | 2.8432 | 4.2548 |  |
| Facility time | 3.3702 | 0.3130 | 2.2574 | 2.7417 | 3.9986 | 6.9804 | 0.4252 | 3.0369 | 6.1263 | 7.8345 |  |
| **HIV+DM** |  |  |  |  |  |  |  |  |  |  |  |
| Travel time | 2.0667 | 0.5254 | 1.6615 | 0.8781 | 3.2552 | 2.1905 | 0.5187 | 1.3724 | 0.9212 | 3.4598 |  |
| Facility time | 1.3833 | 0.6417 | 2.0292 | -0.0683 | 2.8349 | 7.2381 | 4.2017 | 11.1167 | -3.0432 | 17.5194 |  |
| **HTN+DM** |  |  |  |  |  |  |  |  |  |  |  |
| Travel time | 1.5530 | 0.1672 | 1.6719 | 1.2213 | 1.8847 | 2.9346 | 0.1574 | 1.6134 | 2.6224 | 3.2468 |  |
| Facility time | 3.5900 | 0.2919 | 2.8895 | 3.0107 | 4.1693 | 7.2127 | 0.2300 | 2.3565 | 6.7567 | 7.6687 |  |
| **HIV+HTN+DM** |  |  |  |  |  |  |  |  |  |  |  |
| Travel time | 1.9833 | 0.2715 | 0.8587 | 1.3691 | 2.5976 | 2.8500 | 0.3994 | 1.5977 | 1.9987 | 3.7013 |  |
| Facility time | 4.2063 | 0.9319 | 2.6358 | 2.0027 | 6.4098 | 7.5833 | 0.6007 | 2.4029 | 6.3029 | 8.8638 |  |
| **TANZANIA** | | | | | | | | | | |  |
|  | **Integrated care** | | | | | **Standard care** | | | | |  |
| **Health condition** | **Mean** | **Std. Err.** | **Std. Dev.** | **[95% Conf. Interval]** | | **Mean** | **Std. Err.** | **Std. Dev.** | **[95% Conf. Interval]** | |  |
| **HIV ALONE** |  |  |  |  |  |  |  |  |  |  |  |
| Travel time | 1.9857 | 0.0722 | 1.3483 | 1.8437 | 2.1276 | 2.1294 | 0.0690 | 1.3967 | 1.9938 | 2.2650 |  |
| Facility time | 1.4022 | 0.0950 | 1.7573 | 1.2153 | 1.5892 | 1.8258 | 0.0551 | 1.1107 | 1.7175 | 1.9340 |  |
| **HTN ALONE** |  |  |  |  |  |  |  |  |  |  |  |
| Travel time | 1.6827 | 0.1244 | 1.2690 | 1.4359 | 1.9295 | 1.8535 | 0.1034 | 1.0286 | 1.6484 | 2.0587 |  |
| Facility time | 2.0630 | 0.2869 | 2.9259 | 1.4940 | 2.6320 | 2.4948 | 0.1520 | 1.4967 | 2.1932 | 2.7965 |  |
| **DM ALONE** |  |  |  |  |  |  |  |  |  |  |  |
| Travel time | 2.2396 | 0.3773 | 1.5092 | 1.4354 | 3.0438 | 1.7667 | 0.1360 | 0.8046 | 1.4903 | 2.0431 |  |
| Facility time | 1.6563 | 0.1738 | 0.6951 | 1.2858 | 2.0267 | 2.9905 | 0.5728 | 3.3887 | 1.8264 | 4.1546 |  |
| **HIV+HTN** |  |  |  |  |  |  |  |  |  |  |  |
| Travel time | 1.8285 | 0.1736 | 1.2877 | 1.4804 | 2.1766 | 4.2467 | 0.4182 | 2.9568 | 3.4063 | 5.0870 |  |
| Facility time | 1.3524 | 0.1159 | 0.8597 | 1.1200 | 1.5848 | 5.1871 | 1.7898 | 12.5287 | 1.5884 | 8.7857 |  |
| **HIV+DM** |  |  |  |  |  |  |  |  |  |  |  |
| Travel time | 2.2083 | 0.4711 | 1.3326 | 1.0943 | 3.3224 | 3.6000 | 0.5982 | 2.3169 | 2.3169 | 4.8831 |  |
| Facility time | 1.6875 | 0.3528 | 0.9978 | 0.8533 | 2.5217 | 3.7667 | 0.6776 | 2.6245 | 2.3133 | 5.2201 |  |
| **HTN+DM** |  |  |  |  |  |  |  |  |  |  |  |
| Travel time | 2.5667 | 0.2889 | 1.7089 | 1.9796 | 3.1537 | 4.0506 | 0.2331 | 2.0721 | 3.5865 | 4.5148 |  |
| Facility time | 2.2095 | 0.1493 | 0.8832 | 1.9061 | 2.5129 | 5.1961 | 0.3457 | 3.0333 | 4.5076 | 5.8846 |  |
| **HIV+HTN+DM** |  |  |  |  |  |  |  |  |  |  |  |
| Travel time | 1.5208 | 0.4254 | 1.2033 | 0.5149 | 2.5268 | 4.5714 | 0.9221 | 2.4398 | 2.3150 | 6.8278 |  |
| Facility time | 1.3125 | 0.2681 | 0.7582 | 0.6786 | 1.9464 | 5.4286 | 0.9476 | 2.5071 | 3.1099 | 7.7473 |  |

## Table S5. Patient cost sub-sample characteristics, number of participants (percent).

|  | **Uganda** | | **Tanzania** | |
| --- | --- | --- | --- | --- |
|  | **Integrated care**  **(n=637)** | **Standard care**  **(n=801)** | **Integrated care**  **(n=575)** | **Standard care**  **(n=695)** |
| ***Health condition*** | | | | |
| HIV alone | 260 | 376 | 349 | 410 |
|  | (40.82%) | (46.94%) | (60.7%) | (58.99%) |
| HTN alone | 170 | 205 | 104 | 99 |
|  | (26.69%) | (25.59%) | (18.09%) | (14.24%) |
| DM alone | 34 | 41 | 16 | 35 |
|  | (5.34%) | (5.12%) | (2.78%) | (5.04%) |
| HIV+HTN | 52 | 51 | 55 | 50 |
|  | (8.16%) | (6.37%) | (9.57%) | (7.19%) |
| HIV+DM | 10 | 7 | 8 | 15 |
|  | (1.57%) | (0.87%) | (1.39%) | (2.16%) |
| HTN+DM | 101 | 105 | 35 | 79 |
|  | (15.86%) | (13.11%) | (6.09%) | (11.37%) |
| HIV+HTN+DM | 10 | 16 | 8 | 7 |
|  | (1.57%) | (2%) | (1.39%) | (1.01%) |
| ***Sex*** | | | | |
| Female | 500 | 581 | 412 | 520 |
|  | (78.49%) | (72.53%) | (71.65%) | (74.82%) |
| Male | 137 | 220 | 163 | 175 |
|  | (21.51%) | (27.47%) | (28.35%) | (25.18%) |
| ***Education level*** | | | | |
| No formal education | 137 | 117 | 118 | 79 |
|  | (21.51%) | (14.61%) | (20.52%) | (11.37%) |
| Primary school | 362 | 532 | 358 | 485 |
|  | (56.83%) | (66.42%) | (62.26%) | (69.78%) |
| Secondary school or higher | 138 | 152 | 99 | 131 |
|  | (21.66%) | (18.98%) | (17.22%) | (18.85%) |
| ***Age in years*** | | | | |
| <35 | 112 | 136 | 67 | 85 |
|  | (17.58%) | (16.98%) | (11.65%) | (12.23%) |
| 35–49 | 214 | 274 | 226 | 291 |
|  | (33.59%) | (34.21%) | (39.3%) | (41.87%) |
| >=50 | 311 | 391 | 282 | 319 |
|  | (48.82%) | (48.81%) | (49.04%) | (45.9%) |
| ***BMI, kg/m2*** | | | | |
| <25 | 343 | 465 | 253 | 317 |
|  | (53.85%) | (58.05%) | (44%) | (45.61%) |
| 25-29 | 182 | 209 | 163 | 214 |
|  | (28.57%) | (26.09%) | (28.35%) | (30.79%) |
| >=30 | 112 | 127 | 159 | 164 |
|  | (17.58%) | (15.86%) | (27.65%) | (23.6%) |
| ***Blood pressure, mm Hg*** | | | | |
| <140/90 | 406 | 496 | 343 | 423 |
|  | (64.04%) | (62%) | (59.65%) | (60.86%) |
| >=140 or >=90 | 228 | 304 | 232 | 272 |
|  | (35.96%) | (38%) | (40.35%) | (39.14%) |
| ***Fasting blood glucose 6·9 mmol/L among participants with diabetes*** | | | | |
| <7 | 39 | 39 | 10 | 28 |
|  | (33.91%) | (27.46%) | (19.61%) | (23.73%) |
| >=7 | 76 | 103 | 41 | 90 |
|  | (66.09%) | (72.54%) | (80.39%) | (76.27%) |
| ***HIV viral load, copies per mL*** | | | | |
| <1000 | 290 | 401 | 409 | 461 |
|  | (94.46%) | (91.55%) | (97.38%) | (96.24%) |
| >=1000 | 17 | 37 | 11 | 18 |
|  | (5.54%) | (8.45%) | (2.62%) | (3.76%) |
| ***HIV viral load <400 copies per mL*** | | | | |
| <400 | 261 | 334 | 406 | 450 |
|  | (85.01%) | (76.26%) | (96.67%) | (93.95%) |
| >=400 | 46 | 104 | 14 | 29 |
|  | (14.98%) | (23.74%) | (3.33%) | (6.05%) |
| ***Marital status*** | | | | |
| Divorced | 132 | 167 | 100 | 141 |
|  | (20.72%) | (20.85%) | (17.39%) | (20.29%) |
| Married | 311 | 422 | 268 | 309 |
|  | (48.82%) | (52.68%) | (46.61%) | (44.46%) |
| Single | 41 | 58 | 94 | 73 |
|  | (6.44%) | (7.24%) | (16.35%) | (10.5%) |
| Widowed | 153 | 154 | 113 | 172 |
|  | (24.02%) | (19.23%) | (19.65%) | (24.75%) |
| ***Patient is highest earner*** | | | | |
| No | 173 | 284 | 243 | 322 |
|  | (27.16%) | (35.46%) | (42.26%) | (46.33%) |
| Yes | 464 | 517 | 332 | 373 |
|  | (72.84%) | (64.54%) | (57.74%) | (53.67%) |
| ***Employment type, patient*** | | | | |
| Professional | 112 | 56 | 96 | 141 |
|  | (17.58%) | (6.99%) | (16.7%) | (20.29%) |
| Manual | 363 | 507 | 298 | 359 |
|  | (56.99%) | (63.3%) | (51.83%) | (51.65%) |
| Small business owner | 0 | 17 | 1 | 17 |
|  | (0%) | (2.12%) | (0.17%) | (2.45%) |
| Home maker | 78 | 121 | 80 | 97 |
|  | (12.24%) | (15.11%) | (13.91%) | (13.96%) |
| Unemployed | 60 | 71 | 38 | 40 |
|  | (9.42%) | (8.86%) | (6.61%) | (5.76%) |
| Retired | 21 | 26 | 59 | 37 |
|  | (3.3%) | (3.25%) | (10.26%) | (5.32%) |
| Other | 3 | 3 | 3 | 4 |
|  | (0.47%) | (0.37%) | (0.52%) | (0.58%) |
| ***Monthly household income, 2021 Int$*** | | | | |
| <50 | 197 | 277 | 32 | 16 |
|  | (30.93%) | (34.58%) | (5.57%) | (2.3%) |
| 50-150 | 198 | 234 | 111 | 100 |
|  | (31.08%) | (29.21%) | (19.3%) | (14.39%) |
| 150-250 | 148 | 179 | 144 | 182 |
|  | (23.23%) | (22.35%) | (25.04%) | (26.19%) |
| 250-350 | 30 | 29 | 106 | 141 |
|  | (4.71%) | (3.62%) | (18.43%) | (20.29%) |
| >=350 | 64 | 82 | 182 | 256 |
|  | (10.05%) | (10.24%) | (31.65%) | (36.83%) |
| ***Health insurance*** | | | | |
| No | 637 | 801 | 480 | 626 |
|  | (100%) | (100%) | (83.48%) | (90.07%) |
| Yes | 0 | 0 | 95 | 69 |
|  | (0%) | (0%) | (16.52%) | (9.93%) |

## Table S6a. Population size by country

| **Population size (2021)** | **Value** | **Source** |
| --- | --- | --- |
| Tanzania (18+) | 32,000,000 | UN Population Division Data Portal (2) |
| Uganda (18+) | 22,000,000 | UN Population Division Data Portal (2) |
| Tanzania women (30-79) | 9,578,500 | UN Population Division Data Portal (2) |
| Tanzania men (30-79) | 8,800,869 | UN Population Division Data Portal (2) |
| Uganda women (30-79) | 5,794,602 | UN Population Division Data Portal (2) |
| Uganda men (30-79) | 5,289,428 | UN Population Division Data Portal (2) |

## Table S6b. Number of people living with HIV, hypertension and diabetes by country

| **Prevalence Tanzania** | **Value** | **High-bound** | **Low-bound** | **Source** |
| --- | --- | --- | --- | --- |
| HIV (2021) | 1,536,000 | 1,664,000 | 1,472,000 | *UNAIDS 2021 (3)* |
| Hypertension (2021) | 6,110,257 | 7,948,972 | 4,390,620 | *Lancet NCD consortium % prevalence for men and women aged 30-79 (Zhou et al. 2021 supplement pages 38, 45) (4)* |
| Diabetes (2021) | 3,296,000 | 3,488,000 | 1,472,000 | *Diabetes prevalence (%) in adults 20–79 years, Diabetes Atlas 10th Edition (5)* |
| **Prevalence Uganda** | **Value** | **High-bound** | **Low-bound** | **Source** |
| HIV (2021) | 1,232,000 | 1,342,000 | 1,144,000 | *UNAIDS 2021 (3)* |
| Hypertension (2021) | 3,598,803 | 4,841,225 | 2,467,727 | *Lancet NCD consortium % prevalence for men and women aged 30-79 (Zhou et al. 2021 supplement pages 38, 45) (4)* |
| Diabetes (2021) | 792,000 | 1,166,000 | 638,000.0 | *Diabetes prevalence (%) in adults 20–79 years, Diabetes Atlas 10th Edition (5)* |

## Table S6c. Proportion of each health condition from INTE-AFRICA trial data with 95%CIs

| **UGANDA MULTIMORBIDITY DISTRIBUTION (trial data)** | | | | |
| --- | --- | --- | --- | --- |
|  | **Proportion** | **Std. Err.** | **Logit  [95% Conf. Interval]** | |
| HIV alone | 0.442 | 0.009 | 0.426 | 0.459 |
| HTN alone | 0.268 | 0.008 | 0.253 | 0.283 |
| DM alone | 0.057 | 0.004 | 0.049 | 0.065 |
| HIV+HTN | 0.076 | 0.005 | 0.068 | 0.086 |
| HIV+DM | 0.009 | 0.002 | 0.006 | 0.013 |
| HTN+DM | 0.136 | 0.006 | 0.125 | 0.148 |
| HIV+HTN+DM | 0.012 | 0.002 | 0.009 | 0.016 |
| **TANZANIA MULTIMORBIDITY DISTRIBUTION (trial data)** | | | | |
|  | **Proportion** | **Std. Err.** | **Logit  [95% Conf. Interval]** | |
| HIV alone | 0.483 | 0.009 | 0.466 | 0.500 |
| HTN alone | 0.216 | 0.007 | 0.203 | 0.231 |
| DM alone | 0.062 | 0.004 | 0.054 | 0.070 |
| HIV+HTN | 0.098 | 0.005 | 0.088 | 0.109 |
| HIV+DM | 0.019 | 0.002 | 0.015 | 0.024 |
| HTN+DM | 0.104 | 0.005 | 0.094 | 0.115 |
| HIV+HTN+DM | 0.018 | 0.002 | 0.014 | 0.024 |

## Table S6d. Existing and target service coverage levels by country

| **Coverage levels Tanzania** | **Current** | **Source** | **Midpoint** | **Target** | **Source** |
| --- | --- | --- | --- | --- | --- |
| HIV alone | 86% | *UNDAIDS 2021 best estimate (3)* | 91% | 95% | *United Republic of Tanzania Health Sector Strategic Plan July 2021 – June 2026 (HSSP V) (6) /UNAIDS* |
| HTN alone | 15% | *Lancet NCD consortium, mean of % coverage for men and women aged 30-79 (Zhou et al. 2021 supplement pages 38, 45) (4)* | 48% | 80% | *United Republic of Tanzania Health Sector Strategic Plan July 2021 – June 2026 (HSSP V) (6)* |
| DM alone | 47% | *Diabetes Atlas 10th Edition, based on: coverage = (prevalence-number of adults undiagnosed)/prevalence (5)* | 63% | 80% | *United Republic of Tanzania Health Sector Strategic Plan July 2021 – June 2026 (HSSP V) (6)* |
| HIV+HTN | 86% | *Assumed equal to condition with lowest coverage* | 91% | 95% | *Assumed equal to condition with highest target coverage* |
| HIV+DM | 86% | *Assumed equal to condition with lowest coverage* | 91% | 95% | *Assumed equal to coverage of condition with highest target coverage* |
| HTN+DM | 47% | *Assumed equal to diabetes coverage* | 63% | 80% | *Assumed equal to coverage of condition with highest target coverage* |
| HIV+HTN+DM | 86% | *Assumed equal to condition with lowest coverage* | 91% | 95% | *Assumed equal to coverage of condition with highest target coverage* |
| **Coverage levels Uganda** | **Current** | **Source** | **Midpoint** | **Target** | **Source** |
| HIV alone | 82% | *UNDAIDS 2021 best estimate (3)* | 89% | 95% | *Republic of Uganda Ministry of Health Strategic Plan 2020/21 - 2024/25 (7) / UNAIDS 2021 (3)* |
| HTN alone | 18% | *Lancet NCD consortium, mean of % coverage for men and women aged 30-79 (Zhou et al. 2021 supplement pages 38, 45) (4)* | 49% | 80% | *Assumed equal to Tanzania NSP* |
| DM alone | 42% | *Diabetes Atlas 10th Edition, based on: coverage = (prevalence-number of adults undiagnosed)/prevalence (5)* | 61% | 80% | *Assumed equal to Tanzania NSP* |
| HIV+HTN | 82% | *Assumed equal to condition with lowest coverage* | 89% | 95% | *Assumed equal to condition with highest target coverage* |
| HIV+DM | 82% | *Assumed equal to condition with lowest coverage* | 89% | 95% | *Assumed equal to coverage of condition with highest target coverage* |
| HTN+DM | 42% | *Assumed equal to diabetes coverage* | 61% | 80% | *Assumed equal to coverage of condition with highest target coverage* |
| HIV+HTN+DM | 82% | *Assumed equal to condition with lowest coverage* | 89% | 95% | *Assumed equal to coverage of condition with highest target coverage* |

## Table S7. Mean provider costs per patient visit by cost component in Uganda and Tanzania (2021 Int$)

| **UGANDA (2021 Int$)** | | | | | | | | | | | |  |  |
| --- | --- | --- | --- | --- | --- | --- | --- | --- | --- | --- | --- | --- | --- |
|  | **Integrated care** | | | | | **Standard care** | | | | | **Total difference** |  |  |
| **Health condition** | **Mean** | **Std. Err.** | **Std. Dev.** | **[95% Conf. Interval]** | | **Mean** | **Std. Err.** | **Std. Dev.** | **[95% Conf. Interval]** | | **Mean difference in cost (95%CI); p-value*** |  |  |
| **HIV ALONE** |  |  |  |  |  |  |  |  |  |  | 4.19 (-0.42, 8.80); p=0.0746 |  |  |
| Personnel | 24.35 | 0.08 | 2.05 | 24.20 | 24.50 | 24.14 | 0.07 | 1.93 | 24.01 | 24.28 |  |  |  |
| Medication | 71.44 | 1.20 | 32.23 | 69.07 | 73.80 | 73.75 | 1.25 | 34.99 | 71.30 | 76.21 |  |  |  |
| Diagnostics | 52.76 | 0.76 | 20.31 | 51.27 | 54.25 | 54.67 | 0.67 | 18.84 | 53.35 | 56.00 |  |  |  |
| Overheads | 20.30 | 0.06 | 1.67 | 20.18 | 20.43 | 20.47 | 0.06 | 1.58 | 20.36 | 20.58 |  |  |  |
| *Administration* | 13.14 | 0.01 | 0.39 | 13.11 | 13.17 | 13.18 | 0.01 | 0.37 | 13.15 | 13.20 |  |  |  |
| *Equipment and furniture* | 2.02 | 0.04 | 1.09 | 1.94 | 2.10 | 2.13 | 0.04 | 1.03 | 2.06 | 2.20 |  |  |  |
| *Rental space* | 5.14 | 0.01 | 0.19 | 5.13 | 5.16 | 5.16 | 0.01 | 0.18 | 5.15 | 5.17 |  |  |  |
| Total | 168.85 | 1.70 | 45.49 | 165.51 | 172.19 | 173.04 | 1.62 | 45.35 | 169.86 | 176.22 |  |  |  |
| **HTN ALONE** |  |  |  |  |  |  |  |  |  |  | -0.50 (-4.93, 3.93); p=0.8250 |  |  |
| Personnel | 23.21 | 0.34 | 7.50 | 22.55 | 23.88 | 24.51 | 0.33 | 6.82 | 23.85 | 25.17 |  |  |  |
| Medication | 53.42 | 1.59 | 35.09 | 50.31 | 56.54 | 51.12 | 1.35 | 27.50 | 48.47 | 53.76 |  |  |  |
| Diagnostics | 0.00 | 0.00 | 0.00 | 0.00 | 0.00 | 0.00 | 0.00 | 0.00 | 0.00 | 0.00 |  |  |  |
| Overheads | 30.67 | 0.13 | 2.96 | 30.41 | 30.94 | 31.19 | 0.13 | 2.70 | 30.93 | 31.45 |  |  |  |
| *Administration* | 19.10 | 0.26 | 5.82 | 18.58 | 19.61 | 20.10 | 0.26 | 5.30 | 19.59 | 20.61 |  |  |  |
| *Equipment and furniture* | 5.01 | 0.13 | 2.78 | 4.76 | 5.25 | 4.53 | 0.12 | 2.53 | 4.28 | 4.77 |  |  |  |
| *Rental space* | 6.57 | 0.00 | 0.08 | 6.56 | 6.58 | 6.56 | 0.00 | 0.07 | 6.55 | 6.57 |  |  |  |
| Total | 107.31 | 1.72 | 38.13 | 103.93 | 110.70 | 106.81 | 1.38 | 28.08 | 104.11 | 109.52 |  |  |  |
| **DM ALONE** |  |  |  |  |  |  |  |  |  |  | 0.85 (-3.02, 4.73); p=0.6648 |  |  |
| Personnel | 30.80 | 0.41 | 3.66 | 29.98 | 31.62 | 29.33 | 0.57 | 6.10 | 28.20 | 30.47 |  |  |  |
| Medication | 11.62 | 0.97 | 8.66 | 9.68 | 13.56 | 15.16 | 1.11 | 11.85 | 12.95 | 17.37 |  |  |  |
| Diagnostics | 11.06 | 0.19 | 1.73 | 10.68 | 11.45 | 10.24 | 0.23 | 2.41 | 9.79 | 10.69 |  |  |  |
| Overheads | 30.69 | 0.11 | 1.00 | 30.47 | 30.92 | 30.29 | 0.16 | 1.66 | 29.98 | 30.60 |  |  |  |
| *Administration* | 20.84 | 0.22 | 1.97 | 20.40 | 21.28 | 20.05 | 0.31 | 3.28 | 19.44 | 20.66 |  |  |  |
| *Equipment and furniture* | 3.33 | 0.14 | 1.22 | 3.06 | 3.61 | 3.82 | 0.19 | 2.04 | 3.44 | 4.20 |  |  |  |
| *Rental space* | 6.52 | 0.03 | 0.25 | 6.46 | 6.57 | 6.42 | 0.04 | 0.42 | 6.34 | 6.49 |  |  |  |
| Total | 84.17 | 1.05 | 9.30 | 82.09 | 86.26 | 85.03 | 1.47 | 15.63 | 82.11 | 87.94 |  |  |  |
| **HIV+HTN** |  |  |  |  |  |  |  |  |  |  | 53.35 (33.65, 73.06); p<0.0001 |  |  |
| Personnel | 24.05 | 0.14 | 1.87 | 23.77 | 24.33 | 48.30 | 0.56 | 5.13 | 47.20 | 49.41 |  |  |  |
| Medication | 105.90 | 4.85 | 63.95 | 96.33 | 115.46 | 107.40 | 8.46 | 78.02 | 90.57 | 124.23 |  |  |  |
| Diagnostics | 46.11 | 1.62 | 21.43 | 42.90 | 49.31 | 42.87 | 2.02 | 18.64 | 38.85 | 46.89 |  |  |  |
| Overheads | 20.55 | 0.12 | 1.53 | 20.32 | 20.78 | 51.38 | 0.48 | 4.46 | 50.42 | 52.34 |  |  |  |
| *Administration* | 13.20 | 0.03 | 0.36 | 13.14 | 13.25 | 32.90 | 0.64 | 5.92 | 31.62 | 34.18 |  |  |  |
| *Equipment and furniture* | 2.18 | 0.08 | 0.99 | 2.04 | 2.33 | 6.77 | 0.17 | 1.58 | 6.43 | 7.11 |  |  |  |
| *Rental space* | 5.17 | 0.01 | 0.18 | 5.14 | 5.20 | 11.71 | 0.01 | 0.12 | 11.69 | 11.74 |  |  |  |
| Total | 196.60 | 5.12 | 67.51 | 186.50 | 206.70 | 249.95 | 9.76 | 90.03 | 230.54 | 269.37 |  |  |  |
| **HIV+DM** |  |  |  |  |  |  |  |  |  |  |  |  |  |
| Personnel | 23.08 | 0.00 | 0.00 | 23.08 | 23.08 | 48.81 | 1.89 | 7.34 | 44.75 | 52.88 | 51.07 (7.26, 94.88); p=0.0239 |  |  |
| Medication | 87.62 | 9.99 | 38.71 | 66.18 | 109.05 | 102.91 | 14.70 | 56.92 | 71.39 | 134.44 |  |  |  |
| Diagnostics | 63.31 | 5.93 | 22.95 | 50.60 | 76.02 | 46.03 | 6.63 | 25.69 | 31.80 | 60.25 |  |  |  |
| Overheads | 21.34 | 0.00 | 0.00 | 21.34 | 21.34 | 48.67 | 1.17 | 4.52 | 46.17 | 51.17 |  |  |  |
| *Administration* | 13.38 | 0.00 | 0.00 | 13.38 | 13.38 | 30.17 | 1.46 | 5.64 | 27.05 | 33.29 |  |  |  |
| *Equipment and furniture* | 2.70 | 0.00 | 0.00 | 2.70 | 2.70 | 7.37 | 0.52 | 2.00 | 6.26 | 8.47 |  |  |  |
| *Rental space* | 5.26 | 0.00 | 0.00 | 5.26 | 5.26 | 11.13 | 0.23 | 0.88 | 10.65 | 11.62 |  |  |  |
| Total | 195.35 | 14.96 | 57.93 | 163.27 | 227.43 | 246.42 | 15.29 | 59.21 | 213.63 | 279.21 |  |  |  |
| **HTN+DM** |  |  |  |  |  |  |  |  |  |  | 71.72 (65.10, 78.34); p<0.0001 |  |  |
| Personnel | 23.79 | 0.11 | 1.66 | 23.58 | 24.00 | 56.04 | 0.73 | 10.84 | 54.60 | 57.49 |  |  |  |
| Medication | 50.30 | 2.23 | 34.69 | 45.91 | 54.70 | 48.82 | 2.39 | 35.26 | 44.12 | 53.53 |  |  |  |
| Diagnostics | 10.28 | 0.15 | 2.31 | 9.99 | 10.57 | 9.66 | 0.22 | 3.19 | 9.24 | 10.09 |  |  |  |
| Overheads | 20.76 | 0.09 | 1.36 | 20.59 | 20.93 | 62.33 | 0.24 | 3.57 | 61.85 | 62.80 |  |  |  |
| *Administration* | 13.25 | 0.02 | 0.32 | 13.21 | 13.29 | 41.82 | 0.48 | 7.02 | 40.88 | 42.76 |  |  |  |
| *Equipment and furniture* | 2.32 | 0.06 | 0.88 | 2.21 | 2.43 | 7.54 | 0.26 | 3.81 | 7.03 | 8.05 |  |  |  |
| *Rental space* | 5.19 | 0.01 | 0.16 | 5.17 | 5.21 | 12.97 | 0.02 | 0.35 | 12.92 | 13.01 |  |  |  |
| Total | 105.14 | 2.20 | 34.22 | 100.80 | 109.47 | 176.85 | 2.58 | 38.04 | 171.78 | 181.93 |  |  |  |
| **HIV+HTN+DM** |  |  |  |  |  |  |  |  |  |  | 125.37 (82.75, 167.99); p<0.0001 |  |  |
| Personnel | 23.51 | 0.30 | 1.37 | 22.89 | 24.14 | 79.77 | 2.11 | 9.44 | 75.35 | 84.19 |  |  |  |
| Medication | 97.59 | 8.31 | 38.10 | 80.25 | 114.94 | 104.26 | 12.92 | 57.78 | 77.22 | 131.30 |  |  |  |
| Diagnostics | 46.03 | 4.21 | 19.29 | 37.25 | 54.81 | 46.11 | 6.45 | 28.84 | 32.61 | 59.61 |  |  |  |
| Overheads | 20.99 | 0.24 | 1.12 | 20.48 | 21.50 | 83.36 | 1.05 | 4.71 | 81.16 | 85.57 |  |  |  |
| *Administration* | 13.30 | 0.06 | 0.26 | 13.18 | 13.42 | 55.24 | 1.63 | 7.29 | 51.83 | 58.65 |  |  |  |
| *Equipment and furniture* | 2.47 | 0.16 | 0.73 | 2.14 | 2.80 | 9.93 | 0.68 | 3.06 | 8.50 | 11.36 |  |  |  |
| *Rental space* | 5.22 | 0.03 | 0.13 | 5.16 | 5.28 | 18.19 | 0.11 | 0.48 | 17.96 | 18.42 |  |  |  |
| Total | 188.13 | 9.99 | 45.79 | 167.28 | 208.97 | 313.50 | 18.88 | 84.43 | 273.98 | 353.01 |  |  |  |
| **Tanzania (2021 Int$)** | | | | | | | | | | | |  |  |
|  | **Integrated care** | | | | | **Standard care** | | | | | **Total difference** |  |  |
| **Health condition** | **Mean** | **Std. Err.** | **Std. Dev.** | **[95% Conf. Interval]** | | **Mean** | **Std. Err.** | **Std. Dev.** | **[95% Conf. Interval]** | | **Mean difference in cost (95%CI); p-value*** |  | **Mean difference in cost (95%CI); p-value*** |
| **HIV ALONE** |  |  |  |  |  |  |  |  |  |  | 6.54 (1.14, 11.95); p=0.0177 |  |  |
| Personnel | 13.01 | 0.06 | 1.78 | 12.89 | 13.13 | 12.65 | 0.06 | 1.80 | 12.52 | 12.77 |  |  |  |
| Medication | 58.74 | 0.89 | 25.33 | 57.00 | 60.49 | 60.94 | 0.85 | 24.00 | 59.26 | 62.62 |  |  |  |
| Diagnostics | 73.58 | 1.14 | 32.54 | 71.34 | 75.81 | 78.13 | 1.14 | 32.02 | 75.90 | 80.37 |  |  |  |
| Overheads | 6.81 | 0.03 | 0.77 | 6.76 | 6.86 | 6.97 | 0.03 | 0.78 | 6.91 | 7.02 |  |  |  |
| *Administration* | 3.71 | 0.02 | 0.57 | 3.67 | 3.75 | 3.59 | 0.02 | 0.58 | 3.55 | 3.63 |  |  |  |
| *Equipment and furniture* | 0.40 | 0.01 | 0.24 | 0.38 | 0.42 | 0.45 | 0.01 | 0.24 | 0.43 | 0.47 |  |  |  |
| *Rental space* | 2.71 | 0.04 | 1.10 | 2.63 | 2.78 | 2.93 | 0.04 | 1.11 | 2.85 | 3.01 |  |  |  |
| Total | 152.14 | 1.97 | 56.15 | 148.28 | 156.00 | 158.69 | 1.93 | 54.24 | 154.90 | 162.48 |  |  |  |
| **HTN ALONE** |  |  |  |  |  |  |  |  |  |  | 0.39 (-0.97, 1.74); p=0.5754 |  |  |
| Personnel | 9.88 | 0.03 | 0.55 | 9.82 | 9.94 | 10.08 | 0.03 | 0.57 | 10.02 | 10.14 |  |  |  |
| Medication | 9.86 | 0.53 | 9.77 | 8.82 | 10.90 | 10.16 | 0.44 | 8.63 | 9.29 | 11.03 |  |  |  |
| Diagnostics | 0.00 | 0.00 | 0.00 | 0.00 | 0.00 | 0.00 | 0.00 | 0.00 | 0.00 | 0.00 |  |  |  |
| Overheads | 7.44 | 0.02 | 0.30 | 7.41 | 7.47 | 7.33 | 0.02 | 0.31 | 7.30 | 7.36 |  |  |  |
| *Administration* | 4.44 | 0.06 | 1.09 | 4.32 | 4.55 | 4.05 | 0.06 | 1.13 | 3.94 | 4.16 |  |  |  |
| *Equipment and furniture* | 0.15 | 0.01 | 0.18 | 0.13 | 0.17 | 0.21 | 0.01 | 0.18 | 0.19 | 0.23 |  |  |  |
| *Rental space* | 2.85 | 0.03 | 0.61 | 2.79 | 2.92 | 3.07 | 0.03 | 0.63 | 3.01 | 3.13 |  |  |  |
| Total | 27.18 | 0.53 | 9.82 | 26.14 | 28.23 | 27.57 | 0.45 | 8.67 | 26.69 | 28.44 |  |  |  |
| **DM ALONE** |  |  |  |  |  |  |  |  |  |  | 2.50 (0.57, 4.44); p=0.0114 |  |  |
| Personnel | 9.97 | 0.07 | 0.59 | 9.82 | 10.11 | 9.87 | 0.05 | 0.56 | 9.78 | 9.97 |  |  |  |
| Medication | 5.85 | 0.51 | 4.11 | 4.83 | 6.87 | 8.67 | 0.68 | 8.02 | 7.33 | 10.01 |  |  |  |
| Diagnostics | 6.22 | 0.16 | 1.26 | 5.90 | 6.53 | 6.02 | 0.12 | 1.37 | 5.79 | 6.25 |  |  |  |
| Overheads | 7.78 | 0.02 | 0.15 | 7.74 | 7.82 | 7.76 | 0.01 | 0.14 | 7.73 | 7.78 |  |  |  |
| *Administration* | 4.46 | 0.12 | 0.94 | 4.23 | 4.69 | 4.61 | 0.08 | 0.89 | 4.46 | 4.76 |  |  |  |
| *Equipment and furniture* | 0.20 | 0.03 | 0.22 | 0.15 | 0.26 | 0.17 | 0.02 | 0.21 | 0.13 | 0.20 |  |  |  |
| *Rental space* | 3.12 | 0.11 | 0.87 | 2.90 | 3.33 | 2.98 | 0.07 | 0.83 | 2.84 | 3.12 |  |  |  |
| Total | 29.82 | 0.45 | 3.60 | 28.92 | 30.71 | 32.32 | 0.63 | 7.50 | 31.07 | 33.57 |  |  |  |
| **HIV+HTN** |  |  |  |  |  |  |  |  |  |  | 22.41 (9.62, 35.20); p=0.0006 |  |  |
| Personnel | 12.45 | 0.14 | 1.79 | 12.17 | 12.73 | 22.79 | 0.10 | 1.23 | 22.60 | 22.98 |  |  |  |
| Medication | 58.06 | 2.04 | 26.00 | 54.04 | 62.08 | 57.11 | 1.93 | 24.58 | 53.31 | 60.92 |  |  |  |
| Diagnostics | 66.19 | 2.58 | 32.97 | 61.09 | 71.29 | 71.98 | 2.86 | 36.52 | 66.33 | 77.63 |  |  |  |
| Overheads | 7.05 | 0.06 | 0.77 | 6.93 | 7.17 | 14.28 | 0.04 | 0.46 | 14.21 | 14.35 |  |  |  |
| *Administration* | 3.53 | 0.04 | 0.57 | 3.44 | 3.61 | 7.82 | 0.13 | 1.71 | 7.56 | 8.09 |  |  |  |
| *Equipment and furniture* | 0.47 | 0.02 | 0.24 | 0.44 | 0.51 | 0.62 | 0.03 | 0.43 | 0.55 | 0.68 |  |  |  |
| *Rental space* | 3.05 | 0.09 | 1.10 | 2.88 | 3.22 | 5.84 | 0.14 | 1.75 | 5.57 | 6.11 |  |  |  |
| Total | 143.75 | 4.49 | 57.35 | 134.88 | 152.62 | 166.16 | 4.70 | 59.96 | 156.89 | 175.44 |  |  |  |
| **HIV+DM** |  |  |  |  |  |  |  |  |  |  | 26.11 (-11.18, 63.40); p=0.1664 |  |  |
| Personnel | 12.92 | 0.37 | 1.83 | 12.17 | 13.67 | 22.93 | 0.20 | 1.21 | 22.53 | 23.34 |  |  |  |
| Medication | 56.16 | 6.28 | 31.42 | 43.19 | 69.13 | 61.14 | 6.35 | 38.63 | 48.26 | 74.02 |  |  |  |
| Diagnostics | 68.88 | 8.02 | 40.10 | 52.33 | 85.43 | 72.22 | 7.17 | 43.59 | 57.69 | 86.76 |  |  |  |
| Overheads | 6.85 | 0.16 | 0.79 | 6.53 | 7.18 | 14.62 | 0.15 | 0.93 | 14.31 | 14.94 |  |  |  |
| *Administration* | 3.68 | 0.12 | 0.58 | 3.44 | 3.92 | 8.11 | 0.25 | 1.53 | 7.61 | 8.62 |  |  |  |
| *Equipment and furniture* | 0.41 | 0.05 | 0.24 | 0.31 | 0.51 | 0.62 | 0.08 | 0.47 | 0.46 | 0.77 |  |  |  |
| *Rental space* | 2.76 | 0.23 | 1.13 | 2.30 | 3.23 | 5.89 | 0.33 | 1.99 | 5.23 | 6.56 |  |  |  |
| Total | 144.81 | 13.59 | 67.93 | 116.77 | 172.85 | 170.92 | 12.26 | 74.59 | 146.05 | 195.79 |  |  |  |
| **HTN+DM** |  |  |  |  |  |  |  |  |  |  | 16.43 (13.69, 19.17); p<0.0001 |  |  |
| Personnel | 13.56 | 0.16 | 1.59 | 13.24 | 13.88 | 19.50 | 0.06 | 1.00 | 19.37 | 19.62 |  |  |  |
| Medication | 13.69 | 1.17 | 11.64 | 11.37 | 16.01 | 15.11 | 0.78 | 12.29 | 13.57 | 16.65 |  |  |  |
| Diagnostics | 5.90 | 0.17 | 1.68 | 5.57 | 6.24 | 6.31 | 0.08 | 1.29 | 6.15 | 6.47 |  |  |  |
| Overheads | 6.58 | 0.07 | 0.69 | 6.44 | 6.71 | 15.25 | 0.01 | 0.14 | 15.23 | 15.26 |  |  |  |
| *Administration* | 3.88 | 0.05 | 0.51 | 3.78 | 3.98 | 9.51 | 0.11 | 1.78 | 9.29 | 9.74 |  |  |  |
| *Equipment and furniture* | 0.33 | 0.02 | 0.21 | 0.28 | 0.37 | 0.22 | 0.02 | 0.35 | 0.18 | 0.27 |  |  |  |
| *Rental space* | 2.37 | 0.10 | 0.98 | 2.17 | 2.56 | 5.51 | 0.08 | 1.29 | 5.35 | 5.67 |  |  |  |
| Total | 39.73 | 1.11 | 11.07 | 37.52 | 41.94 | 56.16 | 0.76 | 11.95 | 54.67 | 57.65 |  |  |  |
| **HIV+HTN+DM** |  |  |  |  |  |  |  |  |  |  | 57.17 (27.23, 87.10); p=0.0003 |  |  |
| Personnel | 12.64 | 0.35 | 1.83 | 11.91 | 13.36 | 32.84 | 0.11 | 0.64 | 32.62 | 33.06 |  |  |  |
| Medication | 55.61 | 5.18 | 26.91 | 44.97 | 66.26 | 60.39 | 4.87 | 28.41 | 50.48 | 70.31 |  |  |  |
| Diagnostics | 53.04 | 4.73 | 24.58 | 43.32 | 62.77 | 70.13 | 6.71 | 39.15 | 56.47 | 83.79 |  |  |  |
| Overheads | 6.97 | 0.15 | 0.79 | 6.66 | 7.29 | 22.07 | 0.11 | 0.62 | 21.85 | 22.29 |  |  |  |
| *Administration* | 3.59 | 0.11 | 0.59 | 3.35 | 3.82 | 12.21 | 0.46 | 2.68 | 11.27 | 13.14 |  |  |  |
| *Equipment and furniture* | 0.45 | 0.05 | 0.25 | 0.35 | 0.55 | 0.84 | 0.11 | 0.66 | 0.61 | 1.07 |  |  |  |
| *Rental space* | 2.94 | 0.22 | 1.13 | 2.49 | 3.38 | 9.02 | 0.45 | 2.65 | 8.10 | 9.95 |  |  |  |
| Total | 128.27 | 9.17 | 47.62 | 109.43 | 147.11 | 185.43 | 11.16 | 65.07 | 162.73 | 208.14 |  |  |  |

## Table S8. Personnel and overheads sensitivity analyses for mean costs per patient visit (2021 Int$)

| **UGANDA** | | | | | | | | | | | | |
| --- | --- | --- | --- | --- | --- | --- | --- | --- | --- | --- | --- | --- |
| **Base case** | | | | | | | | | | | | |
|  | **Integrated** | | | | **Standard** | | | | **Difference** | | | |
| **Health condition** | **Mean** | **Std. Dev.** | **[95% Conf. Interval]** | | **Mean** | **Std. Dev.** | **[95% Conf. Interval]** | | **Mean** | **[95% Conf. Interval]** | | **p-value** |
| **HIV ALONE** | 168.85 | 45.49 | 165.51 | 172.19 | 173.04 | 45.35 | 169.86 | 176.22 | 4.19 | -0.42 | 8.80 | 0.0746 |
| **HTN ALONE** | 107.31 | 38.13 | 103.93 | 110.70 | 106.81 | 28.08 | 104.11 | 109.52 | -0.50 | -4.93 | 3.93 | 0.825 |
| **DM ALONE** | 84.17 | 9.30 | 82.09 | 86.26 | 85.03 | 15.63 | 82.11 | 87.94 | 0.85 | -3.02 | 4.73 | 0.6648 |
| **HIV+HTN** | 196.60 | 67.51 | 186.50 | 206.70 | 249.95 | 90.03 | 230.54 | 269.37 | 53.35 | 33.65 | 73.06 | 0 |
| **HIV+DM** | 195.35 | 57.93 | 163.27 | 227.43 | 246.42 | 59.21 | 213.63 | 279.21 | 51.07 | 7.26 | 94.88 | 0.0239 |
| **HTN+DM** | 105.14 | 34.22 | 100.80 | 109.47 | 176.85 | 38.04 | 171.78 | 181.93 | 71.72 | 65.10 | 78.34 | 0 |
| **HIV+HTN+DM** | 188.13 | 45.79 | 167.28 | 208.97 | 313.50 | 84.43 | 273.98 | 353.01 | 125.37 | 82.75 | 167.99 | 0 |
| **Staff/overheads +10%** | | | | | | | | | | | | |
|  | **Integrated** | | | | **Standard** | | | | **Difference** | | | |
| **Health condition** | **Mean** | **Std. Dev.** | **[95% Conf. Interval]** | | **Mean** | **Std. Dev.** | **[95% Conf. Interval]** | | **Mean** | **[95% Conf. Interval]** | | **p-value** |
| **HIV ALONE** | 168.85 | 45.49 | 165.51 | 172.19 | 173.04 | 45.35 | 169.86 | 176.22 | 4.19 | -0.42 | 8.80 | 0.0746 |
| **HTN ALONE** | 107.31 | 38.13 | 103.93 | 110.70 | 106.81 | 28.08 | 104.11 | 109.52 | -0.50 | -4.93 | 3.93 | 0.825 |
| **DM ALONE** | 84.17 | 9.30 | 82.09 | 86.26 | 85.03 | 15.63 | 82.11 | 87.94 | 0.85 | -3.02 | 4.73 | 0.6648 |
| **HIV+HTN** | 201.06 | 67.51 | 190.96 | 211.16 | 249.95 | 90.03 | 230.54 | 269.37 | 48.89 | 29.19 | 68.60 | 0 |
| **HIV+DM** | 199.79 | 57.93 | 167.71 | 231.87 | 246.42 | 59.21 | 213.63 | 279.21 | 46.63 | 2.82 | 90.44 | 0.0378 |
| **HTN+DM** | 109.59 | 34.22 | 105.26 | 113.92 | 176.85 | 38.04 | 171.78 | 181.93 | 67.26 | 60.64 | 73.88 | 0 |
| **HIV+HTN+DM** | 192.58 | 45.79 | 171.73 | 213.42 | 313.50 | 84.43 | 273.98 | 353.01 | 120.92 | 78.30 | 163.54 | 0 |
| **Staff/overheads +20%** | | | | | | | | | | | | |
|  | **Integrated** | | | | **Standard** | | | | **Difference** | | | |
| **Health condition** | **Mean** | **Std. Dev.** | **[95% Conf. Interval]** | | **Mean** | **Std. Dev.** | **[95% Conf. Interval]** | | **Mean** | **[95% Conf. Interval]** | | **p-value** |
| **HIV ALONE** | 168.85 | 45.49 | 165.51 | 172.19 | 173.04 | 45.35 | 169.86 | 176.22 | 4.19 | -0.42 | 8.80 | 0.0746 |
| **HTN ALONE** | 107.31 | 38.13 | 103.93 | 110.70 | 106.81 | 28.08 | 104.11 | 109.52 | -0.50 | -4.93 | 3.93 | 0.825 |
| **DM ALONE** | 84.17 | 9.30 | 82.09 | 86.26 | 85.03 | 15.63 | 82.11 | 87.94 | 0.85 | -3.02 | 4.73 | 0.6648 |
| **HIV+HTN** | 205.52 | 67.51 | 195.42 | 215.62 | 249.95 | 90.03 | 230.54 | 269.37 | 44.43 | 24.73 | 64.14 | 0 |
| **HIV+DM** | 204.23 | 57.93 | 172.15 | 236.31 | 246.42 | 59.21 | 213.63 | 279.21 | 42.19 | -1.62 | 86.00 | 0.0585 |
| **HTN+DM** | 114.05 | 34.22 | 109.71 | 118.38 | 176.85 | 38.04 | 171.78 | 181.93 | 62.81 | 56.19 | 69.43 | 0 |
| **HIV+HTN+DM** | 197.03 | 45.79 | 176.18 | 217.87 | 313.50 | 84.43 | 273.98 | 353.01 | 116.47 | 73.85 | 159.09 | 0 |
| **Staff/overheads +50%** | | | | | | | | | | | | |
|  | **Integrated** | | | | **Standard** | | | | **Difference** | | | |
| **Health condition** | **Mean** | **Std. Dev.** | **[95% Conf. Interval]** | | **Mean** | **Std. Dev.** | **[95% Conf. Interval]** | | **Mean** | **[95% Conf. Interval]** | | **p-value** |
| **HIV ALONE** | 168.85 | 45.49 | 165.51 | 172.19 | 173.04 | 45.35 | 169.86 | 176.22 | 4.19 | -0.42 | 8.80 | 0.0746 |
| **HTN ALONE** | 107.31 | 38.13 | 103.93 | 110.70 | 106.81 | 28.08 | 104.11 | 109.52 | -0.50 | -4.93 | 3.93 | 0.825 |
| **DM ALONE** | 84.17 | 9.30 | 82.09 | 86.26 | 85.03 | 15.63 | 82.11 | 87.94 | 0.85 | -3.02 | 4.73 | 0.6648 |
| **HIV+HTN** | 218.90 | 67.50 | 208.80 | 229.00 | 249.95 | 90.03 | 230.54 | 269.37 | 31.05 | 11.35 | 50.76 | 0.0021 |
| **HIV+DM** | 217.56 | 57.93 | 185.48 | 249.64 | 246.42 | 59.21 | 213.63 | 279.21 | 28.86 | -14.95 | 72.67 | 0.188 |
| **HTN+DM** | 127.41 | 34.21 | 123.08 | 131.74 | 176.85 | 38.04 | 171.78 | 181.93 | 49.44 | 42.82 | 56.06 | 0 |
| **HIV+HTN+DM** | 210.38 | 45.80 | 189.53 | 231.22 | 313.50 | 84.43 | 273.98 | 353.01 | 103.12 | 60.50 | 145.74 | 0 |
| **TANZANIA** | | | | | | | | | | | | |
| **Base case** | | | | | | | | | | | | |
|  | **Integrated** | | | | **Standard** | | | | **Difference** | | | |
| **Health condition** | **Mean** | **Std. Dev.** | **[95% Conf. Interval]** | | **Mean** | **Std. Dev.** | **[95% Conf. Interval]** | | **Mean** | **[95% Conf. Interval]** | | **p-value** |
| **HIV ALONE** | 152.14 | 56.15 | 148.28 | 156.00 | 158.69 | 54.24 | 154.90 | 162.48 | 6.54 | 1.14 | 11.95 | 0.0177 |
| **HTN ALONE** | 27.18 | 9.82 | 26.14 | 28.23 | 27.57 | 8.67 | 26.69 | 28.44 | 0.39 | -0.97 | 1.74 | 0.5754 |
| **DM ALONE** | 29.82 | 3.60 | 28.92 | 30.71 | 32.32 | 7.50 | 31.07 | 33.57 | 2.50 | 0.57 | 4.44 | 0.0114 |
| **HIV+HTN** | 143.75 | 57.35 | 134.88 | 152.62 | 166.16 | 59.96 | 156.89 | 175.44 | 22.41 | 9.62 | 35.20 | 0.0006 |
| **HIV+DM** | 144.81 | 67.93 | 116.77 | 172.85 | 170.92 | 74.59 | 146.05 | 195.79 | 26.11 | -11.18 | 63.40 | 0.1664 |
| **HTN+DM** | 39.73 | 11.07 | 37.52 | 41.94 | 56.16 | 11.95 | 54.67 | 57.65 | 16.43 | 13.69 | 19.17 | 0 |
| **HIV+HTN+DM** | 128.27 | 47.62 | 109.43 | 147.11 | 185.43 | 65.07 | 162.73 | 208.14 | 57.17 | 27.23 | 87.10 | 0.0003 |
| **Staff/overheads +10%** | | | | | | | | | | | | |
|  | **Integrated** | | | | **Standard** | | | | **Difference** | | | |
| **Health condition** | **Mean** | **Std. Dev.** | **[95% Conf. Interval]** | | **Mean** | **Std. Dev.** | **[95% Conf. Interval]** | | **Mean** | **[95% Conf. Interval]** | | **p-value** |
| **HIV ALONE** | 152.14 | 56.15 | 148.28 | 156.00 | 158.69 | 54.24 | 154.90 | 162.48 | 6.54 | 1.14 | 11.95 | 0.0177 |
| **HTN ALONE** | 27.18 | 9.82 | 26.14 | 28.23 | 27.57 | 8.67 | 26.69 | 28.44 | 0.39 | -0.97 | 1.74 | 0.5754 |
| **DM ALONE** | 29.82 | 3.60 | 28.92 | 30.71 | 32.32 | 7.50 | 31.07 | 33.57 | 2.50 | 0.57 | 4.44 | 0.0114 |
| **HIV+HTN** | 145.70 | 57.34 | 136.83 | 154.57 | 166.16 | 59.96 | 156.89 | 175.44 | 20.46 | 7.68 | 33.25 | 0.0018 |
| **HIV+DM** | 146.78 | 67.93 | 118.74 | 174.82 | 170.92 | 74.59 | 146.05 | 195.79 | 24.13 | -13.15 | 61.42 | 0.2004 |
| **HTN+DM** | 41.74 | 11.06 | 39.54 | 43.95 | 56.16 | 11.95 | 54.67 | 57.65 | 14.42 | 11.68 | 17.15 | 0 |
| **HIV+HTN+DM** | 130.23 | 47.59 | 111.40 | 149.05 | 185.43 | 65.07 | 162.73 | 208.14 | 55.21 | 25.28 | 85.13 | 0.0005 |
| **Staff/overheads +20%** | | | | | | | | | | | | |
|  | **Integrated** | | | | **Standard** | | | | **Difference** | | | |
| **Health condition** | **Mean** | **Std. Dev.** | **[95% Conf. Interval]** | | **Mean** | **Std. Dev.** | **[95% Conf. Interval]** | | **Mean** | **[95% Conf. Interval]** | | **p-value** |
| **HIV ALONE** | 152.14 | 56.15 | 148.28 | 156.00 | 158.69 | 54.24 | 154.90 | 162.48 | 6.54 | 1.14 | 11.95 | 0.0177 |
| **HTN ALONE** | 27.18 | 9.82 | 26.14 | 28.23 | 27.57 | 8.67 | 26.69 | 28.44 | 0.39 | -0.97 | 1.74 | 0.5754 |
| **DM ALONE** | 29.82 | 3.60 | 28.92 | 30.71 | 32.32 | 7.50 | 31.07 | 33.57 | 2.50 | 0.57 | 4.44 | 0.0114 |
| **HIV+HTN** | 147.65 | 57.33 | 138.79 | 156.52 | 166.16 | 59.96 | 156.89 | 175.44 | 18.51 | 5.73 | 31.29 | 0.0047 |
| **HIV+DM** | 148.76 | 67.93 | 120.72 | 176.80 | 170.92 | 74.59 | 146.05 | 195.79 | 22.16 | -15.13 | 59.44 | 0.2393 |
| **HTN+DM** | 43.75 | 11.04 | 41.55 | 45.96 | 56.16 | 11.95 | 54.67 | 57.65 | 12.40 | 9.67 | 15.14 | 0 |
| **HIV+HTN+DM** | 132.19 | 47.56 | 113.38 | 151.00 | 185.43 | 65.07 | 162.73 | 208.14 | 53.25 | 23.33 | 83.16 | 0.0007 |
| **Staff/overheads +50%** | | | | | | | | | | | | |
|  | **Integrated** | | | | **Standard** | | | | **Difference** | | | |
| **Health condition** | **Mean** | **Std. Dev.** | **[95% Conf. Interval]** | | **Mean** | **Std. Dev.** | **[95% Conf. Interval]** | | **Mean** | **[95% Conf. Interval]** | | **p-value** |
| **HIV ALONE** | 152.14 | 56.15 | 148.28 | 156.00 | 158.69 | 54.24 | 154.90 | 162.48 | 6.54 | 1.14 | 11.95 | 0.0177 |
| **HTN ALONE** | 27.18 | 9.82 | 26.14 | 28.23 | 27.57 | 8.67 | 26.69 | 28.44 | 0.39 | -0.97 | 1.74 | 0.5754 |
| **DM ALONE** | 29.82 | 3.60 | 28.92 | 30.71 | 32.32 | 7.50 | 31.07 | 33.57 | 2.50 | 0.57 | 4.44 | 0.0114 |
| **HIV+HTN** | 153.50 | 57.29 | 144.64 | 162.36 | 166.16 | 59.96 | 156.89 | 175.44 | 12.66 | -0.12 | 25.44 | 0.0522 |
| **HIV+DM** | 154.69 | 67.93 | 126.65 | 182.73 | 170.92 | 74.59 | 146.05 | 195.79 | 16.23 | -21.06 | 53.51 | 0.3875 |
| **HTN+DM** | 49.79 | 10.99 | 47.60 | 51.99 | 56.16 | 11.95 | 54.67 | 57.65 | 6.36 | 3.63 | 9.10 | 0 |
| **HIV+HTN+DM** | 138.07 | 47.46 | 119.30 | 156.84 | 185.43 | 65.07 | 162.73 | 208.14 | 47.36 | 17.46 | 77.26 | 0.0024 |

## Table S9. Mean patient costs per patient visit by cost component in Uganda and Tanzania (2021 Int$)

| **UGANDA (2021 Int$)** | | | | | | | | | | | | |  |
| --- | --- | --- | --- | --- | --- | --- | --- | --- | --- | --- | --- | --- | --- |
|  | **Integrated care** | | | | | **Standard care** | | | | | | **Total difference** |  |
| **Health condition** | **Mean** | **Std. Err.** | **Std. Dev.** | **[95% Conf. Interval]** | | **Mean** | **Std. Err.** | | **Std. Dev.** | **[95% Conf. Interval]** | | **Mean difference in cost (95%CI); p-value*** |  |
| **HIV ALONE** |  |  |  |  |  |  |  |  | |  |  |  |  |
| *Travel time* | *1.9258* | *0.1049* | *1.6053* | *1.7190* | *2.1325* | *1.7262* | *0.0570* | *1.1053* | | *1.6142* | *1.8383* | 0.8422  (-1.4087, 3.0931);  p=0.4627 |  |
| *Facility time* | *2.1104* | *0.1316* | *2.1060* | *1.8512* | *2.3696* | *3.1425* | *0.0937* | *1.8006* | | *2.9582* | *3.3268* |  |  |
| Medical costs | 0.1710 | 0.0832 | 1.3415 | 0.0071 | 0.3348 | 0.2073 | 0.0035 | 0.0678 | | 0.2005 | 0.2142 |  |  |
| Medication | 0.0377 | 0.0181 | 0.4433 | 0.0021 | 0.0732 | 0.0413 | 0.0005 | 0.0124 | | 0.0403 | 0.0422 |  |  |
| Non-medication health expenses | 0.1140 | 0.0753 | 1.2147 | -0.0344 | 0.2623 | 0.1640 | 0.0030 | 0.0575 | | 0.1582 | 0.1698 |  |  |
| Travel | 9.1145 | 0.7279 | 11.7372 | 7.6811 | 10.5479 | 9.4948 | 0.7764 | 15.0550 | | 7.9681 | 11.0214 |  |  |
| Patient productivity | 1.6555 | 0.0791 | 1.2752 | 1.4998 | 1.8113 | 2.0895 | 0.0475 | 0.9214 | | 1.9961 | 2.1830 |  |  |
| Caregiver productivity | 0.0269 | 0.0054 | 0.0875 | 0.0162 | 0.0376 | 0.0185 | 0.0036 | 0.0703 | | 0.0114 | 0.0256 |  |  |
| Total patient | 10.9679 | 0.7797 | 12.5718 | 9.4326 | 12.5032 | 11.8102 | 0.7860 | 15.2407 | | 10.2647 | 13.3556 |  |  |
|  |  |  |  |  |  |  |  |  | |  |  |  |  |
| **HTN ALONE** |  |  |  |  |  |  |  |  | |  |  |  |  |
| *Travel time* | *1.6205* | *0.1025* | *1.3127* | *1.4181* | *1.8229* | *1.7860* | *0.0964* | *1.3809* | | *1.5959* | *1.9762* | -2.0478  (-6.1355, 2.0398);  p=0.3252 |  |
| *Facility time* | *2.9323* | *0.1918* | *2.4261* | *2.5535* | *3.3111* | *3.6594* | *0.0889* | *1.2699* | | *3.4841* | *3.8347* |  |  |
| Medical costs | 10.1721 | 1.3150 | 17.1459 | 7.5761 | 12.7681 | 8.5867 | 0.0310 | 0.4436 | | 8.5257 | 8.6478 |  |  |
| Medication | 8.1813 | 0.5997 | 12.1430 | 7.0024 | 9.3602 | 8.2818 | 0.0142 | 0.2664 | | 8.2539 | 8.3097 |  |  |
| Non-medication health expenses | 0.1101 | 0.0452 | 0.5896 | 0.0208 | 0.1994 | 0.3010 | 0.0198 | 0.2835 | | 0.2620 | 0.3401 |  |  |
| Travel | 9.0501 | 1.3135 | 17.1262 | 6.4571 | 11.6432 | 8.1038 | 0.9930 | 14.2176 | | 6.1459 | 10.0616 |  |  |
| Patient productivity | 1.8780 | 0.0911 | 1.1874 | 1.6982 | 2.0577 | 2.3577 | 0.0595 | 0.8518 | | 2.2404 | 2.4750 |  |  |
| Caregiver productivity | 0.0256 | 0.0067 | 0.0878 | 0.0123 | 0.0389 | 0.0297 | 0.0062 | 0.0889 | | 0.0174 | 0.0419 |  |  |
| Total patient | 21.1257 | 1.9357 | 25.2381 | 17.3045 | 24.9470 | 19.0779 | 1.0039 | 14.3742 | | 17.0985 | 21.0573 |  |  |
|  |  |  |  |  |  |  |  |  | |  |  |  |  |
| **DM ALONE** |  |  |  |  |  |  |  |  | |  |  |  |  |
| *Travel time* | *1.6657* | *0.1583* | *0.9095* | *1.3432* | *1.9881* | *2.1870* | *0.2148* | *1.3755* | | *1.7528* | *2.6211* | 10.5387  (-3.3981, 24.4755); p=0.1361 |  |
| *Facility time* | *1.9436* | *0.3739* | *2.1801* | *1.1830* | *2.7043* | *3.9207* | *0.2178* | *1.3947* | | *3.4805* | *4.3609* |  |  |
| Medical costs | 8.0502 | 2.0761 | 12.1059 | 3.8262 | 12.2741 | 7.8989 | 0.0045 | 0.0287 | | 7.8898 | 7.9079 |  |  |
| Medication | 6.3086 | 1.2160 | 9.4971 | 3.8763 | 8.7409 | 6.3554 | 0.0386 | 0.3765 | | 6.2787 | 6.4321 |  |  |
| Non-medication health expenses | 1.6972 | 0.6807 | 3.9691 | 0.3123 | 3.0821 | 1.5281 | 0.0679 | 0.4347 | | 1.3909 | 1.6653 |  |  |
| Travel | 6.1466 | 1.4293 | 8.3342 | 3.2386 | 9.0545 | 15.7482 | 5.7193 | 36.6214 | | 4.1891 | 27.3074 |  |  |
| Patient productivity | 1.5466 | 0.1890 | 1.1019 | 1.1621 | 1.9310 | 2.6532 | 0.1369 | 0.8768 | | 2.3764 | 2.9299 |  |  |
| Caregiver productivity | 0.0447 | 0.0220 | 0.1285 | -0.0001 | 0.0896 | 0.0265 | 0.0136 | 0.0868 | | -0.0009 | 0.0539 |  |  |
| Total patient | 15.7880 | 3.3063 | 19.2791 | 9.0612 | 22.5148 | 26.3267 | 5.7426 | 36.7708 | | 14.7204 | 37.9330 |  |  |
|  |  |  |  |  |  |  |  |  | |  |  |  |  |
| **HIV+HTN** |  |  |  |  |  |  |  |  | |  |  |  |  |
| *Travel time* | *1.8782* | *0.1700* | *1.2257* | *1.5370* | *2.2194* | *3.5490* | *0.3514* | *2.5094* | | *2.8432* | *4.2548* | 2.8419  (-3.6925, 9.3764); p=0.3903 |  |
| *Facility time* | *3.3702* | *0.3130* | *2.2574* | *2.7417* | *3.9986* | *6.9804* | *0.4252* | *3.0369* | | *6.1263* | *7.8345* |  |  |
| Medical costs | 7.2730 | 2.0031 | 14.4445 | 3.2516 | 11.2944 | 4.7107 | 0.0525 | 0.3746 | | 4.6054 | 4.8161 |  |  |
| Medication | 4.4325 | 0.7460 | 9.1979 | 2.9584 | 5.9065 | 4.4467 | 0.0387 | 0.3352 | | 4.3696 | 4.5238 |  |  |
| Non-medication health expenses | 0.1500 | 0.1231 | 0.8876 | -0.0971 | 0.3971 | 0.2118 | 0.0119 | 0.0853 | | 0.1878 | 0.2358 |  |  |
| Travel | 9.2495 | 0.9877 | 7.1222 | 7.2667 | 11.2323 | 12.2870 | 2.0799 | 14.8533 | | 8.1094 | 16.4646 |  |  |
| Patient productivity | 2.2799 | 0.1511 | 1.0899 | 1.9764 | 2.5833 | 4.5739 | 0.2034 | 1.4528 | | 4.1653 | 4.9825 |  |  |
| Caregiver productivity | 0.0125 | 0.0093 | 0.0668 | -0.0061 | 0.0311 | 0.0852 | 0.0298 | 0.2132 | | 0.0252 | 0.1451 |  |  |
| Total patient | 18.8149 | 2.4817 | 17.8955 | 13.8328 | 23.7970 | 21.6569 | 2.1588 | 15.4171 | | 17.3207 | 25.9930 |  |  |
|  |  |  |  |  |  |  |  |  | |  |  |  |  |
| **HIV+DM** |  |  |  |  |  |  |  |  | |  |  |  |  |
| *Travel time* | *2.0667* | *0.5254* | *1.6615* | *0.8781* | *3.2552* | *2.1905* | *0.5187* | *1.3724* | | *0.9212* | *3.4598* | 18.2430  (-14.9852, 51.4712); p=0.2602 |  |
| *Facility time* | *1.3833* | *0.6417* | *2.0292* | *-0.0683* | *2.8349* | *7.2381* | *4.2017* | *11.1167* | | *-3.0432* | *17.5194* |  |  |
| Medical costs | 4.2888 | 1.8789 | 5.9415 | 0.0385 | 8.5392 | 2.6945 | 0.6957 | 1.8407 | | 0.9922 | 4.3969 |  |  |
| Medication | 3.0635 | 1.4263 | 5.3369 | -0.0180 | 6.1449 | 2.2976 | 0.4000 | 1.3855 | | 1.4173 | 3.1779 |  |  |
| Non-medication health expenses | 0.7798 | 0.7798 | 2.4659 | -0.9842 | 2.5438 | 0.5064 | 0.1307 | 0.3459 | | 0.1864 | 0.8263 |  |  |
| Travel | 11.5409 | 4.1925 | 13.2579 | 2.0567 | 21.0250 | 28.7408 | 15.6175 | 41.3200 | | -9.4739 | 66.9554 |  |  |
| Patient productivity | 1.4987 | 0.3519 | 1.1128 | 0.7026 | 2.2947 | 4.0957 | 1.9552 | 5.1729 | | -0.6884 | 8.8798 |  |  |
| Caregiver productivity | 0.0217 | 0.0217 | 0.0687 | -0.0274 | 0.0709 | 0.0621 | 0.0621 | 0.1642 | | -0.0898 | 0.2139 |  |  |
| Total patient | 17.3501 | 5.8988 | 18.6536 | 4.0061 | 30.6941 | 35.5931 | 16.8178 | 44.4957 | | -5.5586 | 76.7448 |  |  |
|  |  |  |  |  |  |  |  |  | |  |  |  |  |
| **HTN+DM** |  |  |  |  |  |  |  |  | |  |  |  |  |
| *Travel time* | *1.5530* | *0.1672* | *1.6719* | *1.2213* | *1.8847* | *2.9346* | *0.1574* | *1.6134* | | *2.6224* | *3.2468* | 8.4258 (2.4631, 14.3884); p=0.0058 |  |
| *Facility time* | *3.5900* | *0.2919* | *2.8895* | *3.0107* | *4.1693* | *7.2127* | *0.2300* | *2.3565* | | *6.7567* | *7.6687* |  |  |
| Medical costs | 12.5121 | 1.3031 | 13.0957 | 9.9269 | 15.0974 | 13.1971 | 0.1292 | 1.3240 | | 12.9409 | 13.4533 |  |  |
| Medication | 11.3553 | 0.9066 | 12.9168 | 9.5677 | 13.1429 | 11.5135 | 0.0424 | 0.5875 | | 11.4298 | 11.5971 |  |  |
| Non-medication health expenses | 1.1311 | 0.2676 | 2.6891 | 0.6002 | 1.6619 | 1.7441 | 0.0643 | 0.6587 | | 1.6166 | 1.8716 |  |  |
| Travel | 11.1812 | 1.7333 | 17.4196 | 7.7424 | 14.6201 | 16.6652 | 1.8005 | 18.4498 | | 13.0947 | 20.2357 |  |  |
| Patient productivity | 2.1811 | 0.1446 | 1.4534 | 1.8942 | 2.4680 | 4.4079 | 0.1140 | 1.1681 | | 4.1819 | 4.6340 |  |  |
| Caregiver productivity | 0.0280 | 0.0090 | 0.0904 | 0.0101 | 0.0458 | 0.0579 | 0.0167 | 0.1711 | | 0.0248 | 0.0910 |  |  |
| Total patient | 25.9024 | 2.4352 | 24.4735 | 21.0710 | 30.7338 | 34.3282 | 1.8195 | 18.6448 | | 30.7199 | 37.9364 |  |  |
|  |  |  |  |  |  |  |  |  | |  |  |  |  |
| **HIV+HTN+DM** |  |  |  |  |  |  |  |  | |  |  |  |  |
| *Travel time* | *1.9833* | *0.2715* | *0.8587* | *1.3691* | *2.5976* | *2.8500* | *0.3994* | *1.5977* | | *1.9987* | *3.7013* | 2.6558  (-10.7420, 16.0535); p=0.6861 |  |
| *Facility time* | *4.2063* | *0.9319* | *2.6358* | *2.0027* | *6.4098* | *7.5833* | *0.6007* | *2.4029* | | *6.3029* | *8.8638* |  |  |
| Medical costs | 11.6968 | 4.2711 | 13.5063 | 2.0350 | 21.3587 | 10.0824 | 0.1523 | 0.6092 | | 9.7578 | 10.4070 |  |  |
| Medication | 9.9640 | 2.6788 | 11.3651 | 4.3122 | 15.6157 | 10.0993 | 0.1354 | 0.5744 | | 9.8137 | 10.3850 |  |  |
| Non-medication health expenses | 0.0000 | 0.0000 | 0.0000 | 0.0000 | 0.0000 | 0.0000 | 0.0000 | 0.0000 | | 0.0000 | 0.0000 |  |  |
| Travel | 13.4747 | 2.5975 | 8.2142 | 7.5987 | 19.3508 | 15.5958 | 3.5564 | 14.2255 | | 8.0155 | 23.1760 |  |  |
| Patient productivity | 2.3233 | 0.4903 | 1.5505 | 1.2141 | 3.4325 | 4.5322 | 0.3076 | 1.2303 | | 3.8766 | 5.1878 |  |  |
| Caregiver productivity | 0.0869 | 0.0579 | 0.1832 | -0.0441 | 0.2179 | 0.0271 | 0.0271 | 0.1086 | | -0.0307 | 0.0850 |  |  |
| Total patient | 27.5817 | 5.8557 | 18.5175 | 14.3351 | 40.8283 | 30.2375 | 3.6157 | 14.4628 | | 22.5309 | 37.9442 |  |  |
| **TANZANIA (2021 Int$)** | | | | | | | | | | | | |  |
|  | **Integrated care** | | | | | **Standard care** | | | | | | **Total difference** |  |
| **Health condition** | **Mean** | **Std. Err.** | **Std. Dev.** | **[95% Conf. Interval]** | | **Mean** | **Std. Err.** | **Std. Dev.** | | **[95% Conf. Interval]** | | **Mean difference in cost (95%CI); p-value*** |  |
| **HIV ALONE** |  |  |  |  |  |  |  |  | |  |  |  |  |
| *Travel time* | *1.9857* | *0.0722* | *1.3483* | *1.8437* | *2.1276* | *2.1294* | *0.0690* | *1.3967* | | *1.9938* | *2.2650* | 1.2051  (-0.0664, 2.4765); p=0.0632 |  |
| *Facility time* | *1.4022* | *0.0950* | *1.7573* | *1.2153* | *1.5892* | *1.8258* | *0.0551* | *1.1107* | | *1.7175* | *1.9340* |  |  |
| Medical costs | 0.1167 | 0.0599 | 1.1188 | -0.0011 | 0.2345 | 0.4948 | 0.0338 | 0.6838 | | 0.4284 | 0.5612 |  |  |
| Medication | 0.0549 | 0.0255 | 0.7148 | 0.0048 | 0.1050 | 0.0254 | 0.0009 | 0.0249 | | 0.0236 | 0.0272 |  |  |
| Non-medication health expenses | 0.0334 | 0.0334 | 0.6231 | -0.0322 | 0.0990 | 0.4708 | 0.0345 | 0.6977 | | 0.4031 | 0.5385 |  |  |
| Travel | 4.6729 | 0.3066 | 5.7280 | 4.0698 | 5.2759 | 5.2345 | 0.5182 | 10.4938 | | 4.2157 | 6.2533 |  |  |
| Patient productivity | 1.5780 | 0.0550 | 1.0277 | 1.4698 | 1.6862 | 1.8514 | 0.0401 | 0.8118 | | 1.7726 | 1.9302 |  |  |
| Caregiver productivity | 0.0114 | 0.0070 | 0.1309 | -0.0023 | 0.0252 | 0.0034 | 0.0016 | 0.0327 | | 0.0003 | 0.0066 |  |  |
| Total patient | 6.3791 | 0.3307 | 6.1771 | 5.7287 | 7.0294 | 7.5841 | 0.5271 | 10.6727 | | 6.5480 | 8.6203 |  |  |
|  |  |  |  |  |  |  |  |  | |  |  |  |  |
| **HTN ALONE** |  |  |  |  |  |  |  |  | |  |  |  |  |
| *Travel time* | *1.6827* | *0.1244* | *1.2690* | *1.4359* | *1.9295* | *1.8535* | *0.1034* | *1.0286* | | *1.6484* | *2.0587* | -2.2810  (-8.6561, 4.0941); p=0.4813 |  |
| *Facility time* | *2.0630* | *0.2869* | *2.9259* | *1.4940* | *2.6320* | *2.4948* | *0.1520* | *1.4967* | | *2.1932* | *2.7965* |  |  |
| Medical costs | 10.2917 | 2.5791 | 26.3018 | 5.1767 | 15.4068 | 8.1634 | 0.9116 | 9.0704 | | 6.3543 | 9.9725 |  |  |
| Medication | 6.7416 | 1.0329 | 17.0344 | 4.7082 | 8.7751 | 6.3760 | 0.3413 | 6.2281 | | 5.7046 | 7.0474 |  |  |
| Non-medication health expenses | 0.9514 | 0.3414 | 3.4818 | 0.2743 | 1.6285 | 0.9258 | 0.0743 | 0.7392 | | 0.7784 | 1.0732 |  |  |
| Travel | 5.2494 | 0.9380 | 9.5653 | 3.3892 | 7.1096 | 4.8372 | 0.9337 | 9.2900 | | 2.9844 | 6.6901 |  |  |
| Patient productivity | 1.7593 | 0.1447 | 1.4753 | 1.4724 | 2.0462 | 2.0187 | 0.0851 | 0.8464 | | 1.8499 | 2.1875 |  |  |
| Caregiver productivity | 0.0000 | 0.0000 | 0.0000 | 0.0000 | 0.0000 | 0.0000 | 0.0000 | 0.0000 | | 0.0000 | 0.0000 |  |  |
| Total patient | 17.3004 | 2.9104 | 29.6803 | 11.5283 | 23.0725 | 15.0194 | 1.2772 | 12.7084 | | 12.4847 | 17.5540 |  |  |
|  |  |  |  |  |  |  |  |  | |  |  |  |  |
| **DM ALONE** |  |  |  |  |  |  |  |  | |  |  |  |  |
| *Travel time* | *2.2396* | *0.3773* | *1.5092* | *1.4354* | *3.0438* | *1.7667* | *0.1360* | *0.8046* | | *1.4903* | *2.0431* | -2.6383  (-9.0785, 3.8019); p=0.4144 |  |
| *Facility time* | *1.6563* | *0.1738* | *0.6951* | *1.2858* | *2.0267* | *2.9905* | *0.5728* | *3.3887* | | *1.8264* | *4.1546* |  |  |
| Medical costs | 8.1629 | 2.5790 | 10.3161 | 2.6658 | 13.6599 | 9.4363 | 1.5265 | 9.0310 | | 6.3341 | 12.5386 |  |  |
| Medication | 8.4724 | 1.8663 | 13.3284 | 4.7237 | 12.2211 | 6.2144 | 0.8902 | 9.1648 | | 4.4494 | 7.9794 |  |  |
| Non-medication health expenses | 2.0880 | 0.9808 | 3.9230 | -0.0024 | 4.1784 | 3.5054 | 0.2212 | 1.3088 | | 3.0559 | 3.9550 |  |  |
| Travel | 8.1483 | 2.3900 | 9.5601 | 3.0541 | 13.2425 | 3.7648 | 0.5257 | 3.1099 | | 2.6966 | 4.8331 |  |  |
| Patient productivity | 1.8298 | 0.1976 | 0.7905 | 1.4086 | 2.2511 | 2.2344 | 0.2681 | 1.5859 | | 1.6896 | 2.7791 |  |  |
| Caregiver productivity | 0.0000 | 0.0000 | 0.0000 | 0.0000 | 0.0000 | 0.0671 | 0.0671 | 0.3970 | | -0.0693 | 0.2035 |  |  |
| Total patient | 18.1410 | 3.1760 | 12.7040 | 11.3715 | 24.9105 | 15.5026 | 1.6153 | 9.5563 | | 12.2199 | 18.7854 |  |  |
|  |  |  |  |  |  |  |  |  | |  |  |  |  |
| **HIV+HTN** |  |  |  |  |  |  |  |  | |  |  |  |  |
| *Travel time* | *1.8285* | *0.1736* | *1.2877* | *1.4804* | *2.1766* | *4.2467* | *0.4182* | *2.9568* | | *3.4063* | *5.0870* | 6.5970 (2.0267, 11.1673); p=0.0051 |  |
| *Facility time* | *1.3524* | *0.1159* | *0.8597* | *1.1200* | *1.5848* | *5.1871* | *1.7898* | *12.5287* | | *1.5884* | *8.7857* |  |  |
| Medical costs | 3.7355 | 1.1123 | 8.2491 | 1.5055 | 5.9656 | 3.3651 | 0.3181 | 2.2495 | | 2.7258 | 4.0044 |  |  |
| Medication | 2.9027 | 0.5929 | 7.4284 | 1.7316 | 4.0737 | 2.3296 | 0.1940 | 2.3992 | | 1.9464 | 2.7128 |  |  |
| Non-medication health expenses | 0.1058 | 0.1058 | 0.7848 | -0.1063 | 0.3180 | 0.9555 | 0.1210 | 0.8556 | | 0.7123 | 1.1986 |  |  |
| Travel | 5.2424 | 0.9458 | 7.0146 | 3.3461 | 7.1387 | 9.3123 | 1.2934 | 9.1455 | | 6.7132 | 11.9115 |  |  |
| Patient productivity | 1.4940 | 0.1044 | 0.7741 | 1.2848 | 1.7033 | 4.3822 | 0.9122 | 6.4500 | | 2.5491 | 6.2152 |  |  |
| Caregiver productivity | 0.0000 | 0.0000 | 0.0000 | 0.0000 | 0.0000 | 0.0094 | 0.0094 | 0.0664 | | -0.0095 | 0.0283 |  |  |
| Total patient | 10.4720 | 1.4587 | 10.8183 | 7.5474 | 13.3966 | 17.0690 | 1.8077 | 12.7821 | | 13.4363 | 20.7016 |  |  |
|  |  |  |  |  |  |  |  |  | |  |  |  |  |
| **HIV+DM** |  |  |  |  |  |  |  |  | |  |  |  |  |
| *Travel time* | *2.2083* | *0.4711* | *1.3326* | *1.0943* | *3.3224* | *3.6000* | *0.5982* | *2.3169* | | *2.3169* | *4.8831* | 7.9539  (-4.8030, 20.7108); p=0.2088 |  |
| *Facility time* | *1.6875* | *0.3528* | *0.9978* | *0.8533* | *2.5217* | *3.7667* | *0.6776* | *2.6245* | | *2.3133* | *5.2201* |  |  |
| Medical costs | 1.1640 | 1.1640 | 3.2924 | -1.5885 | 3.9166 | 2.0595 | 0.6677 | 2.5859 | | 0.6274 | 3.4915 |  |  |
| Medication | 0.0506 | 0.0506 | 0.2427 | -0.0543 | 0.1556 | 0.4785 | 0.3018 | 1.7072 | | -0.1370 | 1.0940 |  |  |
| Non-medication health expenses | 1.0185 | 1.0185 | 2.8809 | -1.3899 | 3.4270 | 1.5521 | 0.3880 | 1.5028 | | 0.7198 | 2.3843 |  |  |
| Travel | 6.9261 | 1.9724 | 5.5787 | 2.2621 | 11.5900 | 12.3544 | 3.8371 | 14.8611 | | 4.1246 | 20.5842 |  |  |
| Patient productivity | 1.8298 | 0.2906 | 0.8220 | 1.1426 | 2.5170 | 3.4600 | 0.4623 | 1.7905 | | 2.4685 | 4.4516 |  |  |
| Caregiver productivity | 0.0000 | 0.0000 | 0.0000 | 0.0000 | 0.0000 | 0.0000 | 0.0000 | 0.0000 | | 0.0000 | 0.0000 |  |  |
| Total patient | 9.9199 | 2.2388 | 6.3322 | 4.6261 | 15.2137 | 17.8739 | 4.2774 | 16.5663 | | 8.6998 | 27.0480 |  |  |
|  |  |  |  |  |  |  |  |  | |  |  |  |  |
| **HTN+DM** |  |  |  |  |  |  |  |  | |  |  |  |  |
| *Travel time* | *2.5667* | *0.2889* | *1.7089* | *1.9796* | *3.1537* | *4.0506* | *0.2331* | *2.0721* | | *3.5865* | *4.5148* | -3.0198  (-12.6862, 6.6466); p=0.5372 |  |
| *Facility time* | *2.2095* | *0.1493* | *0.8832* | *1.9061* | *2.5129* | *5.1961* | *0.3457* | *3.0333* | | *4.5076* | *5.8846* |  |  |
| Medical costs | 16.8786 | 4.4605 | 26.3888 | 7.8138 | 25.9435 | 12.0743 | 1.6926 | 15.0445 | | 8.7045 | 15.4441 |  |  |
| Medication | 10.6661 | 2.0882 | 19.0241 | 6.5121 | 14.8202 | 6.3714 | 0.7839 | 11.2237 | | 4.8258 | 7.9170 |  |  |
| Non-medication health expenses | 2.2616 | 0.8126 | 4.8073 | 0.6102 | 3.9129 | 3.2635 | 0.1486 | 1.3210 | | 2.9676 | 3.5594 |  |  |
| Travel | 11.3744 | 2.4982 | 14.7796 | 6.2974 | 16.4513 | 11.1277 | 1.3392 | 11.9027 | | 8.4616 | 13.7937 |  |  |
| Patient productivity | 2.2433 | 0.1557 | 0.9213 | 1.9268 | 2.5598 | 4.2813 | 0.1811 | 1.6092 | | 3.9208 | 4.6417 |  |  |
| Caregiver productivity | 0.0067 | 0.0067 | 0.0397 | -0.0069 | 0.0203 | 0.0000 | 0.0000 | 0.0000 | | 0.0000 | 0.0000 |  |  |
| Total patient | 30.5030 | 5.3969 | 31.9283 | 19.5353 | 41.4708 | 27.4832 | 2.2063 | 19.6102 | | 23.0908 | 31.8757 |  |  |
|  |  |  |  |  |  |  |  |  | |  |  |  |  |
| **HIV+HTN+DM** |  |  |  |  |  |  |  |  | |  |  |  |  |
| *Travel time* | *1.5208* | *0.4254* | *1.2033* | *0.5149* | *2.5268* | *4.5714* | *0.9221* | *2.4398* | | *2.3150* | *6.8278* | 9.1284  (-6.3950, 24.6517); p=0.2262 |  |
| *Facility time* | *1.3125* | *0.2681* | *0.7582* | *0.6786* | *1.9464* | *5.4286* | *0.9476* | *2.5071* | | *3.1099* | *7.7473* |  |  |
| Medical costs | 5.4565 | 3.6661 | 10.3693 | -3.2125 | 14.1254 | 12.9814 | 2.8264 | 7.4780 | | 6.0654 | 19.8975 |  |  |
| Medication | 8.7734 | 4.6809 | 24.3225 | -0.8482 | 18.3951 | 8.2623 | 1.4220 | 8.1685 | | 5.3658 | 11.1587 |  |  |
| Non-medication health expenses | 1.6006 | 1.3003 | 3.6777 | -1.4741 | 4.6752 | 3.3813 | 1.1955 | 3.1629 | | 0.4561 | 6.3065 |  |  |
| Travel | 10.4764 | 4.9527 | 14.0084 | -1.2349 | 22.1877 | 8.7137 | 2.5416 | 6.7244 | | 2.4946 | 14.9328 |  |  |
| Patient productivity | 1.3308 | 0.2600 | 0.7355 | 0.7159 | 1.9457 | 4.6969 | 0.3399 | 0.8994 | | 3.8651 | 5.5286 |  |  |
| Caregiver productivity | 0.0000 | 0.0000 | 0.0000 | 0.0000 | 0.0000 | 0.0000 | 0.0000 | 0.0000 | | 0.0000 | 0.0000 |  |  |
| Total patient | 17.2636 | 5.6031 | 15.8480 | 4.0144 | 30.5128 | 26.3920 | 4.2194 | 11.1635 | | 16.0675 | 36.7165 |  |  |

## Table S10. Sensitivity analysis for patient costs per visit, assuming 22 working days per month to value time loss (2021 Int$)

| **Mean patient costs per patient visit** | | | | | | |  |
| --- | --- | --- | --- | --- | --- | --- | --- |
|  | **UGANDA** | | | **TANZANIA** | | |  |
|  |  |  |  |  |  |  |  |
|  | **Integrated care** | **Standard care** | **Mean difference in cost (95%CI); p-value*** | **Integrated care** | **Standard care** | **Mean difference in cost (95%CI); p-value*** |  |
|  | **Mean (SD)** | **Mean (SD)** |  | **Mean (SD)** | **Mean (SD)** |  |  |
| **HIV ALONE** | 11.58 (12.81) | 12.58 (15.31) | 0.99 (-1.28, 3.27); p=0.3893 | 6.96 (6.34) | 8.26 (10.74) | 1.30 (0.02, 2.59); p=0.0471 |  |
| **HTN ALONE** | 21.82 (25.36) | 19.95 (14.41) | -1.87 (-5.98, 2.23); p=0.3706 | 17.94 (29.80) | 15.75 (12.82) | -2.19 (-8.59, 4.22); p=0.5016 |  |
| **DM ALONE** | 16.37 (19.58) | 27.30 (36.83) | 10.93 (-3.06, 24.93); p=0.1238 | 18.81 (12.83) | 16.34 (9.68) | -2.47 (-8.98, 4.05); p=0.4502 |  |
| **HIV+HTN** | 19.65 (18.10) | 23.35 (15.62) | 3.70 (-2.91, 10.32); p=0.2695 | 11.02 (10.92) | 18.67 (14.50) | 7.65 (2.71, 12.59); p=0.0027 |  |
| **HIV+DM** | 17.90 (18.90) | 37.11 (45.47) | 19.20 (-14.69, 53.10); p=0.2459 | 10.59 (6.48) | 19.13 (16.73) | 8.55 (-4.35, 21.44); p=0.1826 |  |
| **HTN+DM** | 26.71 (24.72) | 35.95 (18.65) | 9.25 (3.24, 15.25); p=0.0027 | 31.32 (32.04) | 29.04 (19.74) | -2.28 (-12.00, 7.43); p=0.6427 |  |
| **HIV+HTN+DM** | 28.46 (18.78) | 31.90 (14.53) | 3.44 (-10.09, 16.96); p=0.6047 | 17.75 (15.94) | 28.10 (11.33) | 10.35 (-5.30, 26.01); p=0.1767 |  |

SD=Standard Deviation

*Mean difference in costs, two-sided test at a 5% significance level

## Table S11. Sensitivity analysis for patient costs per visit, using salaries to value time loss (2021 Int$)

| **Mean patient costs per patient visit** | | | | | | |  |
| --- | --- | --- | --- | --- | --- | --- | --- |
|  | **UGANDA** | | | **TANZANIA** | | |  |
|  |  |  |  |  |  |  |  |
|  | **Integrated care** | **Standard care** | **Mean difference in cost (95%CI); p-value*** | **Integrated care** | **Standard care** | **Mean difference in cost (95%CI); p-value*** |  |
|  | **Mean (SD)** | **Mean (SD)** |  | **Mean (SD)** | **Mean (SD)** |  |  |
| **HIV ALONE** | 12.36 (13.14) | 13.56 (15.43) | 1.20 (-1.11, 3.50); p=0.3083 | 8.43 (6.83) | 9.98 (10.94) | 1.55 (0.22, 2.87); p=0.0223 |  |
| **HTN ALONE** | 22.71 (25.54) | 21.06 (14.49) | -1.65 (-5.78, 2.49); p=0.4338 | 19.57 (30.14) | 17.63 (13.13) | -1.95 (-8.44, 4.55); p=0.5551 |  |
| **DM ALONE** | 17.11 (19.96) | 28.55 (36.91) | 11.44 (-2.63, 25.51); p=0.1094 | 20.50 (13.16) | 18.47 (10.13) | -2.03 (-8.79, 4.73); p=0.5492 |  |
| **HIV+HTN** | 20.72 (18.37) | 25.52 (15.91) | 4.81 (-1.92, 11.53); p=0.1593 | 12.40 (11.21) | 22.74 (19.49) | 10.34 (4.25, 16.42); p=0.0011 |  |
| **HIV+DM** | 18.61 (19.23) | 39.04 (46.80) | 20.43 (-14.37, 55.23); p=0.2300 | 12.28 (6.89) | 22.34 (17.26) | 10.06 (-3.27, 23.39); p=0.1315 |  |
| **HTN+DM** | 27.74 (25.06) | 38.03 (18.68) | 10.30 (4.24, 16.35); p=0.0010 | 33.41 (32.34) | 33.01 (20.15) | -0.40 (-10.25, 9.46); p=0.9365 |  |
| **HIV+HTN+DM** | 29.58 (19.14) | 34.02 (14.64) | 4.44 (-9.27, 18.14); p=0.5102 | 18.98 (16.19) | 32.46 (11.77) | 13.47 (-2.54, 29.49); p=0.0922 |  |

SD=Standard Deviation

*Mean difference in costs, two-sided test at a 5% significance level

Note: Median monthly salaries were sourced from the Uganda National Labour Force Survey(8) and from the Integrated Labour Force Survey in Tanzania (9).

## Table S12. Sensitivity analyses for costs at scale (2021 Int$)

| **UGANDA** | | | | | | | | | |
| --- | --- | --- | --- | --- | --- | --- | --- | --- | --- |
| **Current coverage** | | | | | | | | | |
|  | **Integrated care** | | | **Standard care** | | | **Difference (Integrated - Standard)** | | |
|  | **Value** | **HB** | **LB** | **Value** | **HB** | **LB** | **Value** | **HB** | **LB** |
| HIV alone | 668,995,477 | 741,951,796 | 609,936,674 | 698,801,557 | 775,332,135 | 636,835,543 | -29,806,079 | -33,380,339 | -26,898,869 |
| HTN alone | 347,416,601 | 478,718,755 | 218,877,905 | 354,755,964 | 492,836,765 | 221,566,990 | -7,339,363 | -14,118,010 | -2,689,085 |
| DM alone | 61,292,465 | 103,412,508 | 43,583,782 | 51,949,839 | 87,329,033 | 37,102,181 | 9,342,627 | 16,083,475 | 6,481,601 |
| HIV+HTN | 139,322,728 | 156,957,322 | 124,942,509 | 207,122,763 | 241,953,384 | 178,401,056 | -67,800,034 | -84,996,062 | -53,458,547 |
| HIV+DM | 17,929,185 | 20,928,197 | 15,456,627 | 22,435,573 | 30,831,780 | 15,383,241 | -4,506,389 | -9,903,583 | 73,386 |
| HTN+DM | 148,242,221 | 226,651,239 | 114,817,952 | 258,103,172 | 401,054,595 | 196,387,984 | -109,860,952 | -174,403,356 | -81,570,032 |
| HIV+HTN+DM | 28,812,121 | 35,392,150 | 23,337,870 | 47,366,642 | 60,036,250 | 36,788,179 | -18,554,521 | -24,644,100 | -13,450,309 |
| **Total** | **1,412,010,798** | **1,764,011,966** | **1,150,953,320** | **1,640,535,510** | **2,089,373,942** | **1,322,465,174** | **-228,524,712** | **-325,361,975** | **-171,511,854** |
| **% of GDP** | **1.2%** | **1.6%** | **1.0%** | **1.4%** | **1.8%** | **1.2%** | **-0.2%** | **-0.3%** | **-0.2%** |
| **% of CHE** | **32.6%** | **40.7%** | **26.6%** | **37.9%** | **48.2%** | **30.5%** | **-5.3%** | **-7.5%** | **-4.0%** |
| **Midpoint coverage** | | | | | | | | | |
|  | **Integrated care** | | | **Standard care** | | | **Difference (Integrated - Standard)** | | |
|  | **Value** | **HB** | **LB** | **Value** | **HB** | **LB** | **Value** | **HB** | **LB** |
| HIV alone | 722,025,607 | 800,765,048 | 658,285,313 | 754,194,363 | 836,791,390 | 687,316,409 | -32,168,756 | -36,026,342 | -29,031,096 |
| HTN alone | 952,232,896 | 1,312,118,491 | 599,921,654 | 972,349,329 | 1,350,814,495 | 607,292,157 | -20,116,433 | -38,696,003 | -7,370,503 |
| DM alone | 88,485,317 | 149,292,225 | 62,920,046 | 74,997,766 | 126,073,198 | 53,562,836 | 13,487,551 | 23,219,027 | 9,357,211 |
| HIV+HTN | 150,366,603 | 169,399,061 | 134,846,489 | 223,541,030 | 261,132,616 | 192,542,603 | -73,174,427 | -91,733,555 | -57,696,114 |
| HIV+DM | 19,350,401 | 22,587,139 | 16,681,848 | 24,214,003 | 33,275,763 | 16,602,645 | -4,863,602 | -10,688,623 | 79,203 |
| HTN+DM | 214,010,968 | 327,206,723 | 165,757,778 | 372,612,537 | 578,985,406 | 283,516,953 | -158,601,569 | -251,778,683 | -117,759,174 |
| HIV+HTN+DM | 28,812,121 | 35,392,150 | 23,337,870 | 47,366,642 | 60,036,250 | 36,788,179 | -18,554,521 | -24,644,100 | -13,450,309 |
| **Total** | **2,175,283,913** | **2,816,760,838** | **1,661,750,998** | **2,469,275,670** | **3,247,109,117** | **1,877,621,781** | **-293,991,757** | **-430,348,279** | **-215,870,783** |
| **% of GDP** | **1.9%** | **2.5%** | **1.5%** | **2.2%** | **2.9%** | **1.7%** | **-0.3%** | **-0.4%** | **-0.2%** |
| **% of CHE** | **50.2%** | **65.0%** | **38.3%** | **57.0%** | **74.9%** | **43.3%** | **-6.8%** | **-9.9%** | **-5.0%** |
| **Target coverage** | | | | | | | | | |
|  | **Integrated care** | | | **Standard care** | | | **Difference (Integrated - Standard)** | | |
|  | **Value** | **HB** | **LB** | **Value** | **HB** | **LB** | **Value** | **HB** | **LB** |
| HIV alone | 775,055,736 | 859,578,300 | 706,633,952 | 809,587,170 | 898,250,645 | 737,797,275 | -34,531,434 | -38,672,344 | -31,163,323 |
| HTN alone | 1,557,049,192 | 2,145,518,228 | 980,965,402 | 1,589,942,694 | 2,208,792,225 | 993,017,323 | -32,893,503 | -63,273,996 | -12,051,921 |
| DM alone | 115,678,169 | 195,171,943 | 82,256,311 | 98,045,692 | 164,817,364 | 70,023,490 | 17,632,476 | 30,354,579 | 12,232,821 |
| HIV+HTN | 161,410,478 | 181,840,800 | 144,750,468 | 239,959,298 | 280,311,847 | 206,684,150 | -78,548,820 | -98,471,048 | -61,933,682 |
| HIV+DM | 20,771,616 | 24,246,082 | 17,907,068 | 25,992,433 | 35,719,745 | 17,822,048 | -5,220,816 | -11,473,663 | 85,020 |
| HTN+DM | 279,779,716 | 427,762,207 | 216,697,605 | 487,121,902 | 756,916,217 | 370,645,921 | -207,342,185 | -329,154,010 | -153,948,317 |
| HIV+HTN+DM | 33,379,896 | 41,003,100 | 27,037,776 | 54,875,988 | 69,554,192 | 42,620,451 | -21,496,092 | -28,551,091 | -15,582,675 |
| **Total** | **2,943,124,803** | **3,875,120,660** | **2,176,248,582** | **3,305,525,176** | **4,414,362,235** | **2,438,610,659** | **-362,400,373** | **-539,241,575** | **-262,362,078** |
| **% of GDP** | **2.6%** | **3.4%** | **1.9%** | **2.9%** | **3.9%** | **2.2%** | **-0.3%** | **-0.5%** | **-0.2%** |
| **% of CHE** | **67.9%** | **89.4%** | **50.2%** | **76.3%** | **101.9%** | **56.3%** | **-8.4%** | **-12.4%** | **-6.1%** |
| **TANZANIA** | | | | | | | | | |
| **Current coverage** | | | | | | | | | |
|  | **Integrated care** | | | **Standard care** | | | **Difference (Integrated - Standard)** | | |
|  | **Value** | **HB** | **LB** | **Value** | **HB** | **LB** | **Value** | **HB** | **LB** |
| HIV alone | 744,234,202 | 814,764,474 | 705,695,817 | 738,480,466 | 809,179,781 | 699,608,009 | 5,753,736 | 5,584,692 | 6,087,808 |
| HTN alone | 140,432,057 | 206,575,999 | 109,778,834 | 124,044,720 | 182,544,729 | 96,925,658 | 16,387,337 | 24,031,269 | 12,853,176 |
| DM alone | 169,257,387 | 197,628,237 | 62,934,449 | 143,454,269 | 164,606,272 | 54,477,815 | 25,803,119 | 33,021,965 | 8,456,634 |
| HIV+HTN | 157,440,559 | 174,973,803 | 146,976,578 | 183,461,692 | 204,743,187 | 170,515,936 | -26,021,133 | -29,769,384 | -23,539,359 |
| HIV+DM | 30,088,514 | 35,178,083 | 26,550,578 | 34,742,322 | 41,609,849 | 29,780,733 | -4,653,808 | -6,431,766 | -3,230,155 |
| HTN+DM | 275,251,488 | 310,956,422 | 114,626,382 | 355,161,469 | 396,223,091 | 150,018,231 | -79,909,981 | -85,266,669 | -35,391,849 |
| HIV+HTN+DM | 33,546,797 | 39,048,992 | 29,754,689 | 42,952,128 | 51,302,125 | 36,942,253 | -9,405,330 | -12,253,133 | -7,187,564 |
| **Total** | **1,550,251,004** | **1,779,126,010** | **1,196,317,326** | **1,622,297,064** | **1,850,209,036** | **1,238,268,636** | **-72,046,061** | **-71,083,026** | **-41,951,310** |
| **% of GDP** | **0.89%** | **1.02%** | **0.68%** | **0.93%** | **1.06%** | **0.71%** | **-0.04%** | **-0.04%** | **-0.02%** |
| **% of CHE** | **23.13%** | **26.54%** | **17.85%** | **24.20%** | **27.60%** | **18.47%** | **-1.07%** | **-1.06%** | **-0.63%** |
| **Midpoint coverage** | | | | | | | | | |
|  | **Integrated care** | | | **Standard care** | | | **Difference (Integrated - Standard)** | | |
|  | **Value** | **HB** | **LB** | **Value** | **HB** | **LB** | **Value** | **HB** | **LB** |
| HIV alone | 783,176,689 | 857,397,498 | 742,621,761 | 777,121,885 | 851,520,584 | 736,215,405 | 6,054,804 | 5,876,915 | 6,406,356 |
| HTN alone | 440,993,737 | 648,703,177 | 344,734,523 | 389,533,171 | 573,238,647 | 304,372,156 | 51,460,565 | 75,464,530 | 40,362,367 |
| DM alone | 229,509,099 | 267,979,314 | 85,337,656 | 194,520,667 | 223,202,295 | 73,870,656 | 34,988,432 | 44,777,019 | 11,467,000 |
| HIV+HTN | 165,678,728 | 184,129,409 | 154,667,212 | 193,061,432 | 215,456,493 | 179,438,282 | -27,382,704 | -31,327,084 | -24,771,070 |
| HIV+DM | 31,662,913 | 37,018,797 | 27,939,852 | 36,560,234 | 43,787,109 | 31,339,027 | -4,897,321 | -6,768,312 | -3,399,175 |
| HTN+DM | 373,234,645 | 421,649,710 | 155,430,720 | 481,590,730 | 537,269,339 | 203,421,248 | -108,356,085 | -115,619,629 | -47,990,528 |
| HIV+HTN+DM | 35,302,153 | 41,092,254 | 31,311,620 | 45,199,623 | 53,986,539 | 38,875,278 | -9,897,470 | -12,894,285 | -7,563,658 |
| **Total** | **2,059,557,963** | **2,457,970,159** | **1,542,043,345** | **2,117,587,742** | **2,498,461,006** | **1,567,532,053** | **-58,029,779** | **-40,490,847** | **-25,488,708** |
| **% of GDP** | **1.18%** | **1.40%** | **0.88%** | **1.21%** | **1.43%** | **0.90%** | **-0.03%** | **-0.02%** | **-0.01%** |
| **% of CHE** | **30.72%** | **36.67%** | **23.00%** | **31.59%** | **37.27%** | **23.38%** | **-0.87%** | **-0.60%** | **-0.38%** |
| **Target coverage** | | | | | | | | | |
|  | **Integrated care** | | | **Standard care** | | | **Difference (Integrated - Standard)** | | |
|  | **Value** | **HB** | **LB** | **Value** | **HB** | **LB** | **Value** | **HB** | **LB** |
| HIV alone | 822,119,176 | 900,030,523 | 779,547,705 | 815,763,305 | 893,861,386 | 772,822,801 | 6,355,871 | 6,169,137 | 6,724,904 |
| HTN alone | 741,555,416 | 1,090,830,356 | 579,690,212 | 655,021,623 | 963,932,565 | 511,818,654 | 86,533,794 | 126,897,791 | 67,871,558 |
| DM alone | 289,760,811 | 338,330,391 | 107,740,863 | 245,587,066 | 281,798,317 | 93,263,497 | 44,173,745 | 56,532,074 | 14,477,365 |
| HIV+HTN | 173,916,897 | 193,285,015 | 162,357,847 | 202,661,171 | 226,169,800 | 188,360,627 | -28,744,275 | -32,884,785 | -26,002,780 |
| HIV+DM | 33,237,312 | 38,859,510 | 29,329,126 | 38,378,146 | 45,964,369 | 32,897,321 | -5,140,835 | -7,104,858 | -3,568,195 |
| HTN+DM | 471,217,802 | 532,342,997 | 196,235,058 | 608,019,990 | 678,315,587 | 256,824,266 | -136,802,188 | -145,972,589 | -60,589,207 |
| HIV+HTN+DM | 37,057,509 | 43,135,515 | 32,868,551 | 47,447,118 | 56,670,952 | 40,808,303 | -10,389,609 | -13,535,437 | -7,939,751 |
| **Total** | **2,568,864,922** | **3,136,814,308** | **1,887,769,364** | **2,612,878,419** | **3,146,712,975** | **1,896,795,470** | **-44,013,497** | **-9,898,667** | **-9,026,106** |
| **% of GDP** | **1.47%** | **1.79%** | **1.08%** | **1.49%** | **1.80%** | **1.08%** | **-0.03%** | **-0.01%** | **-0.01%** |
| **% of CHE** | **38.32%** | **46.79%** | **28.16%** | **38.98%** | **46.94%** | **28.29%** | **-0.66%** | **-0.15%** | **-0.13%** |

## Table S13. Total provider costs at target levels of service coverage (2021 Int$)

|  | **UGANDA (Midpoint coverage)** | | | **TANZANIA (Midpoint coverage)** | | |
| --- | --- | --- | --- | --- | --- | --- |
|  | **Integrated care** | **Standard care** | **Difference (Integrated–- Standard)** | **Integrated care** | **Standard care** | **Difference (Integrated–- Standard)** |
|  | **Total cost** | **Total cost** | **Difference** | **Total cost** | **Total cost** | **Difference** |
| HIV alone | 722,025,607 | 754,194,363 | -32,168,756 | 783,176,689 | 777,121,885 | 6,054,804 |
| HTN alone | 952,232,896 | 972,349,329 | -20,116,433 | 440,993,737 | 389,533,171 | 51,460,565 |
| DM alone | 88,485,317 | 74,997,766 | 13,487,551 | 229,509,099 | 194,520,667 | 34,988,432 |
| HIV+HTN | 150,366,603 | 223,541,030 | -73,174,427 | 165,678,728 | 193,061,432 | -27,382,704 |
| HIV+DM | 19,350,401 | 24,214,003 | -4,863,602 | 31,662,913 | 36,560,234 | -4,897,321 |
| HTN+DM | 214,010,968 | 372,612,537 | -158,601,569 | 373,234,645 | 481,590,730 | -108,356,085 |
| HIV+HTN+DM | 28,812,121 | 47,366,642 | -18,554,521 | 35,302,153 | 45,199,623 | -9,897,470 |
| **Total** | **2,175,283,913** | **2,469,275,670** | **-293,991,757** | **2,059,557,963** | **2,117,587,742** | **-58,029,779** |
| **% of GDP** | **1.90%** | **2.20%** | **-0.30%** | **1.18%** | **1.21%** | **-0.03%** |
| **% of CHE** | **50.20%** | **57.00%** | **-6.80%** | **30.72%** | **31.59%** | **-0.87%** |
|  | **UGANDA (Target coverage)** | | | **TANZANIA (Target coverage)** | | |
|  | **Integrated care** | **Standard care** | **Difference (Integrated–- Standard)** | **Integrated care** | **Standard care** | **Difference (Integrated–- Standard)** |
|  | **Total cost** | **Total cost** | **Difference** | **Total cost** | **Total cost** | **Difference** |
| HIV alone | 775,055,736 | 809,587,170 | -34,531,434 | 822,119,176 | 815,763,305 | 6,355,871 |
| HTN alone | 1,557,049,192 | 1,589,942,694 | -32,893,503 | 741,555,416 | 655,021,623 | 86,533,794 |
| DM alone | 115,678,169 | 98,045,692 | 17,632,476 | 289,760,811 | 245,587,066 | 44,173,745 |
| HIV+HTN | 161,410,478 | 239,959,298 | -78,548,820 | 173,916,897 | 202,661,171 | -28,744,275 |
| HIV+DM | 20,771,616 | 25,992,433 | -5,220,816 | 33,237,312 | 38,378,146 | -5,140,835 |
| HTN+DM | 279,779,716 | 487,121,902 | -207,342,185 | 471,217,802 | 608,019,990 | -136,802,188 |
| HIV+HTN+DM | 33,379,896 | 54,875,988 | -21,496,092 | 37,057,509 | 47,447,118 | -10,389,609 |
| **Total** | **2,943,124,803** | **3,305,525,176** | **-362,400,373** | **2,568,864,922** | **2,612,878,419** | **-44,013,497** |
| **% of GDP** | **2.60%** | **2.90%** | **-0.30%** | **1.47%** | **1.49%** | **-0.03%** |
| **% of CHE** | **67.90%** | **76.30%** | **-8.40%** | **38.32%** | **38.98%** | **-0.66%** |

## Figure S1. Mean provider costs per visit for patients receiving integrated or standard care, by cost component


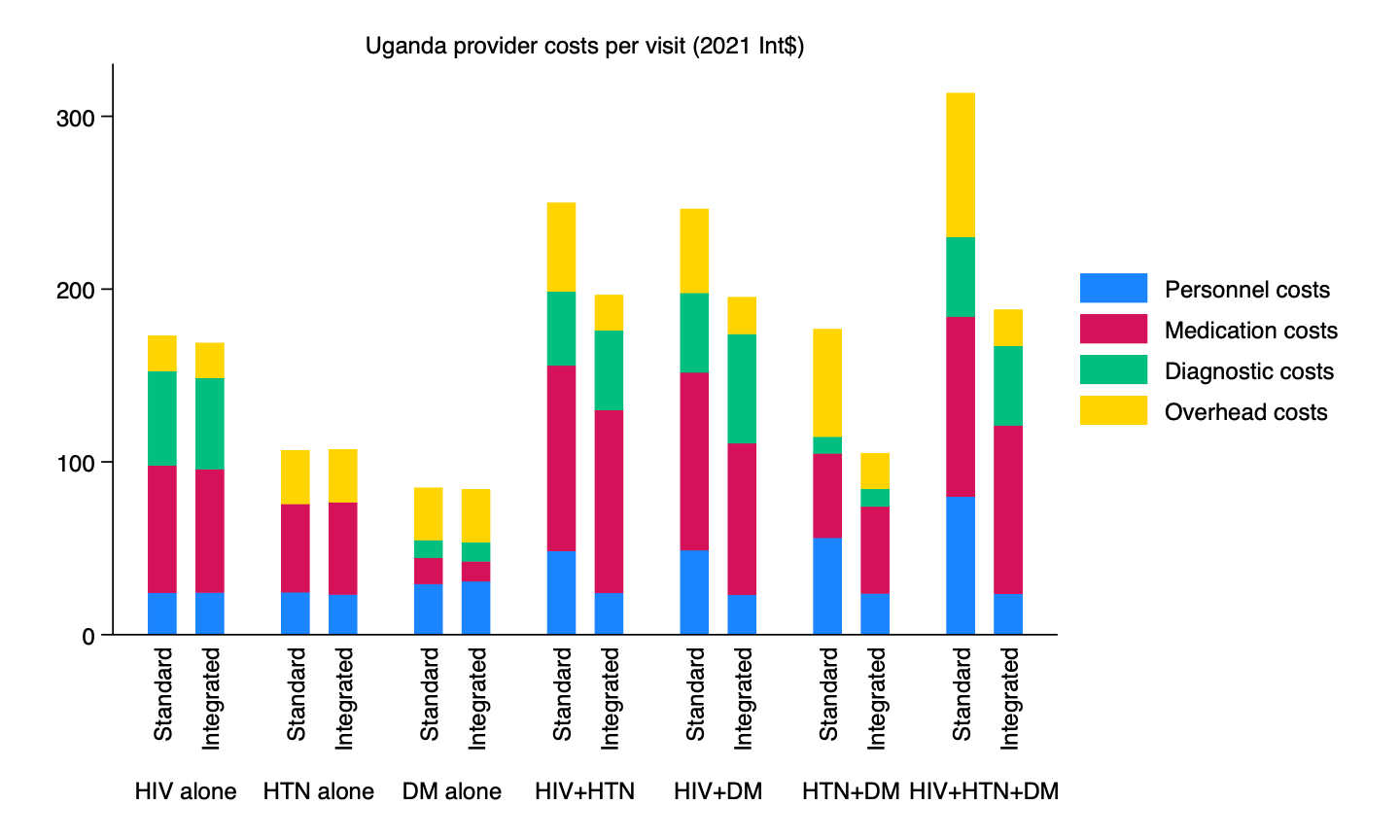


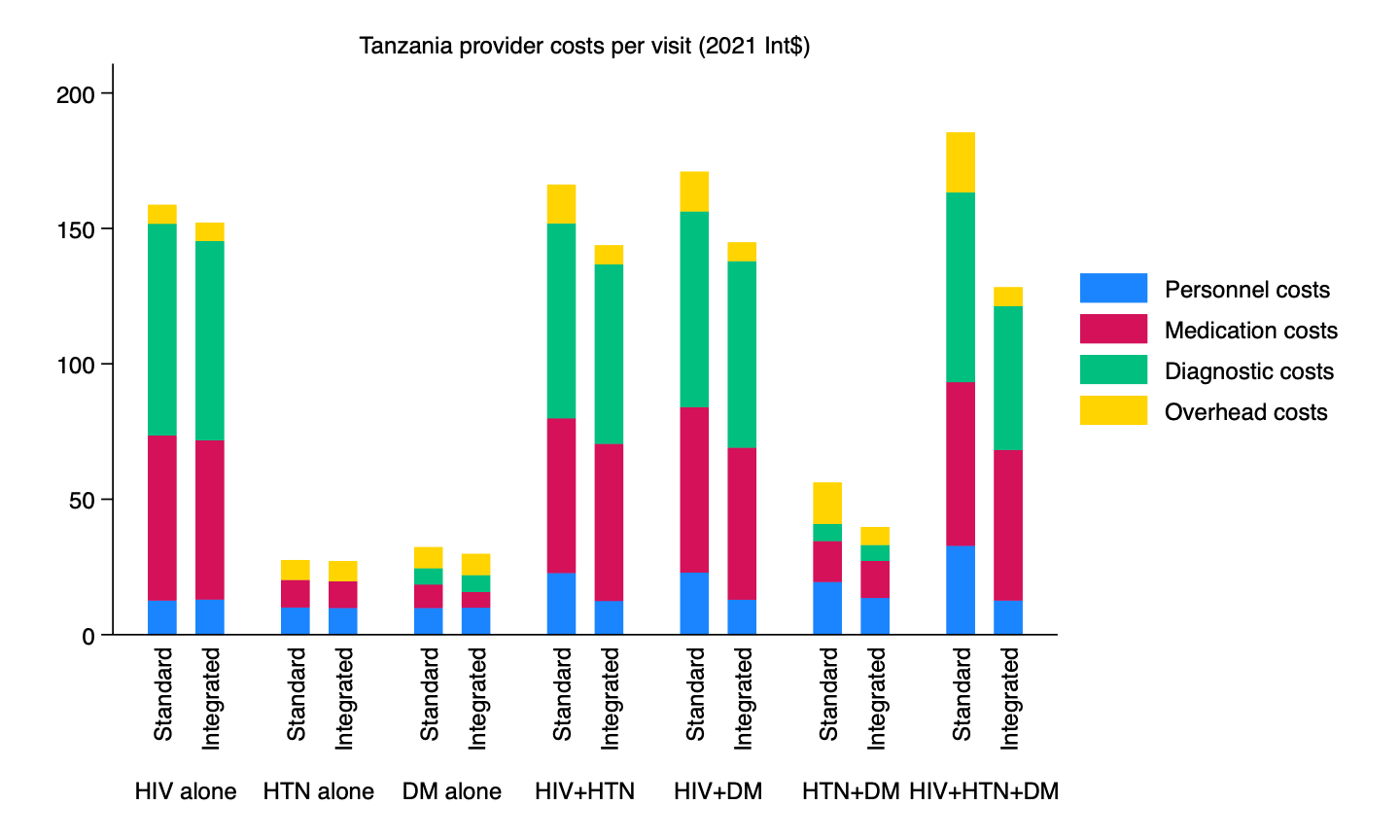


## Figure S2. Mean provider costs per patient visit by health facility in Uganda and Tanzania (2021 Int$)

**
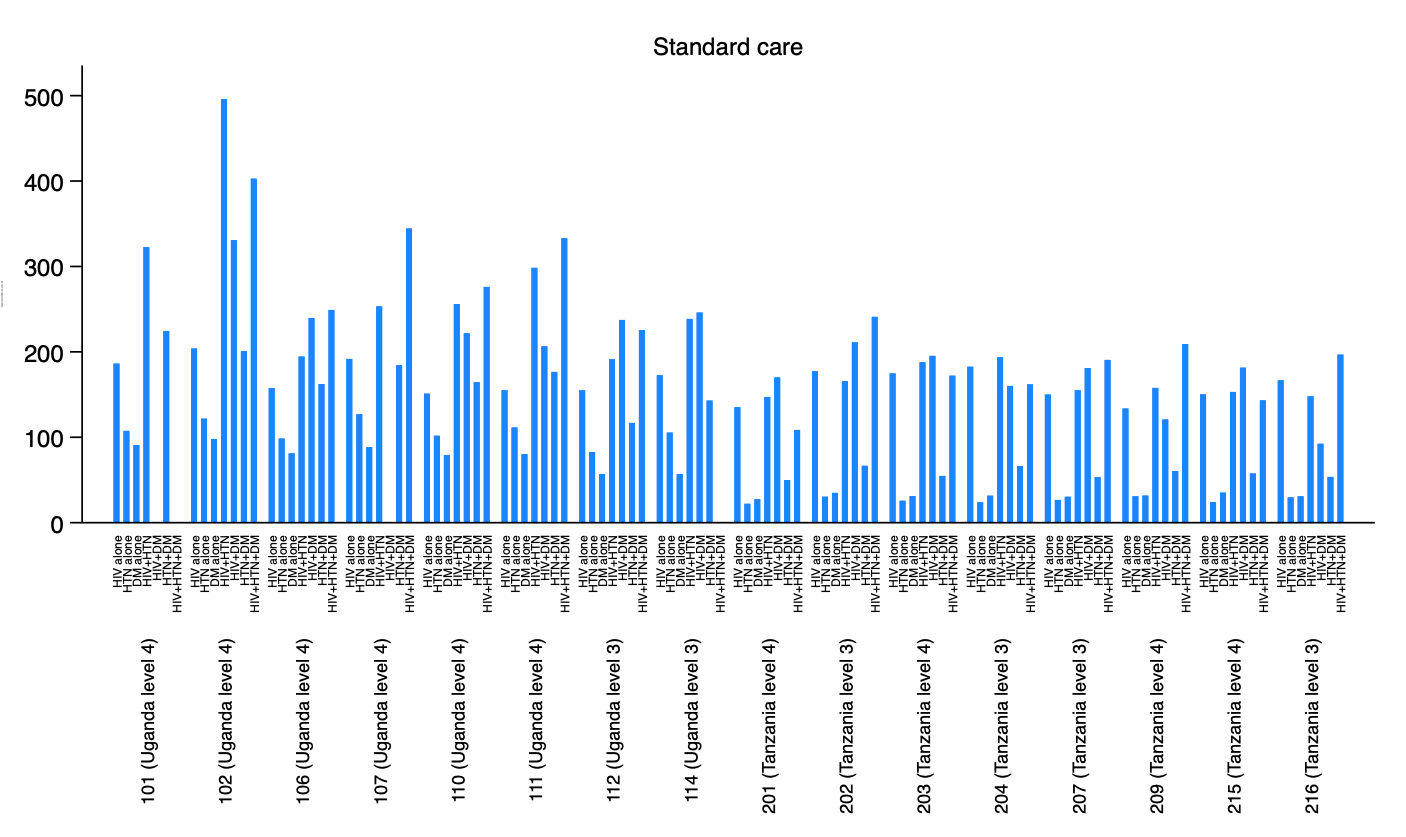
**

**
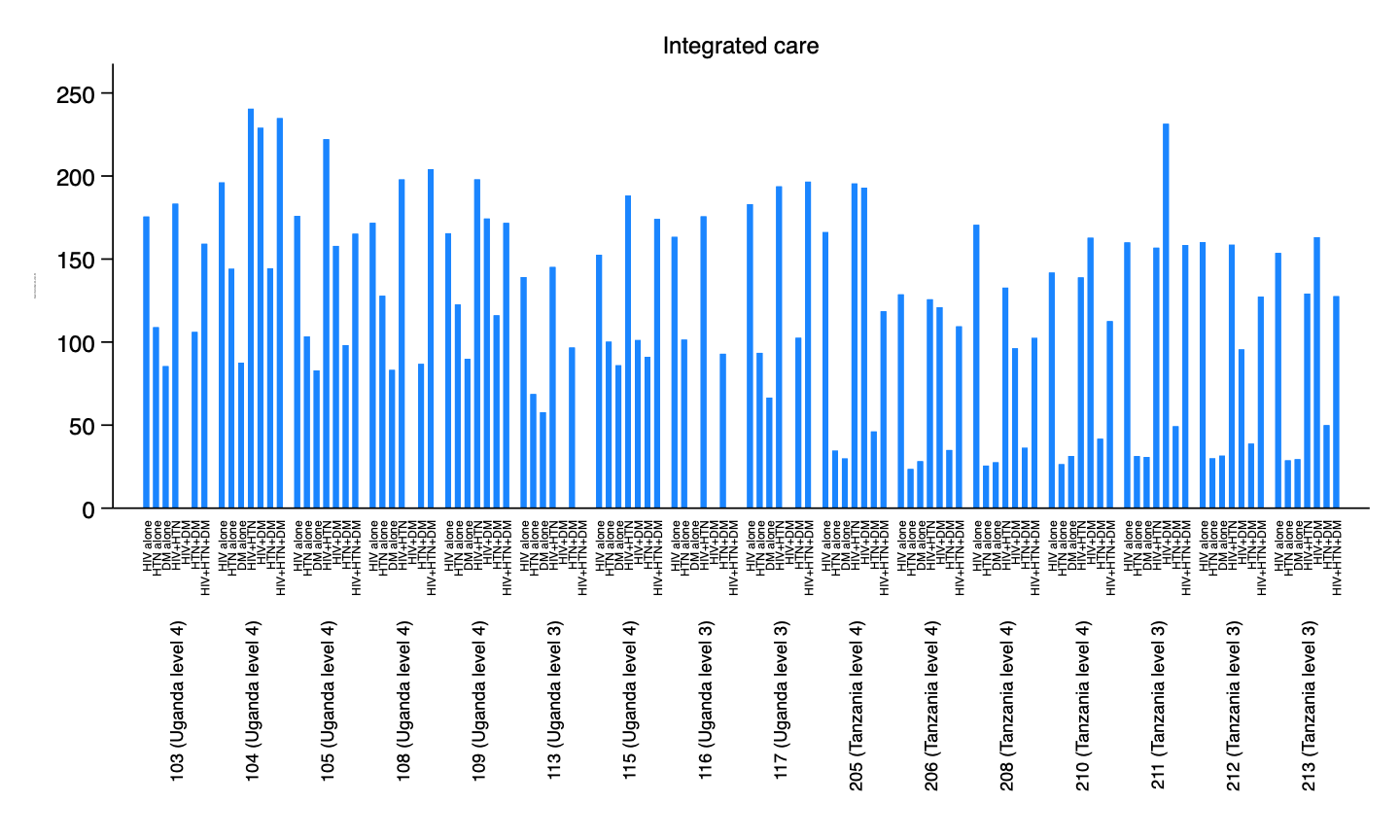
**

## Figure S3. Mean patient costs per visit for patients receiving integrated or vertical care, by cost component

**
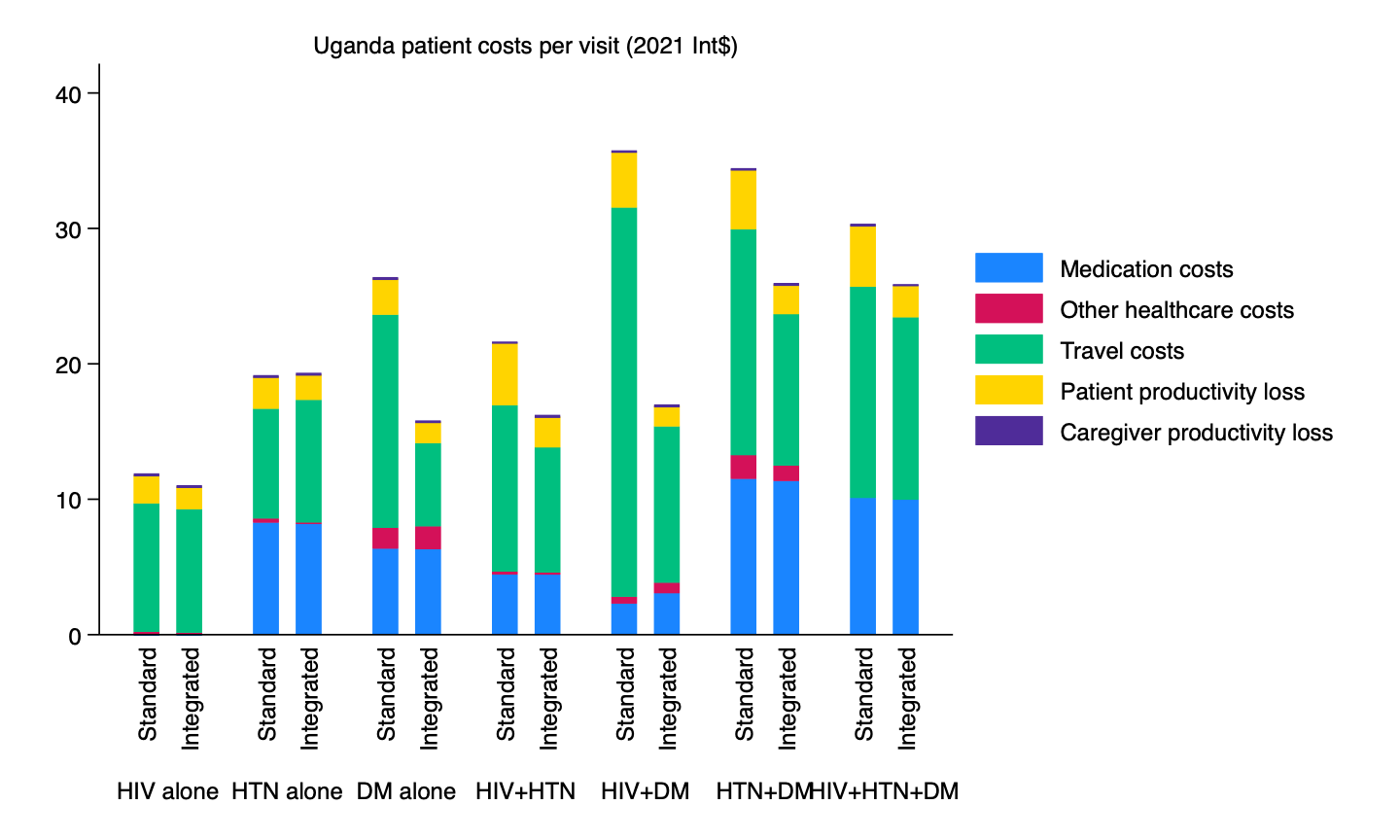
**

**
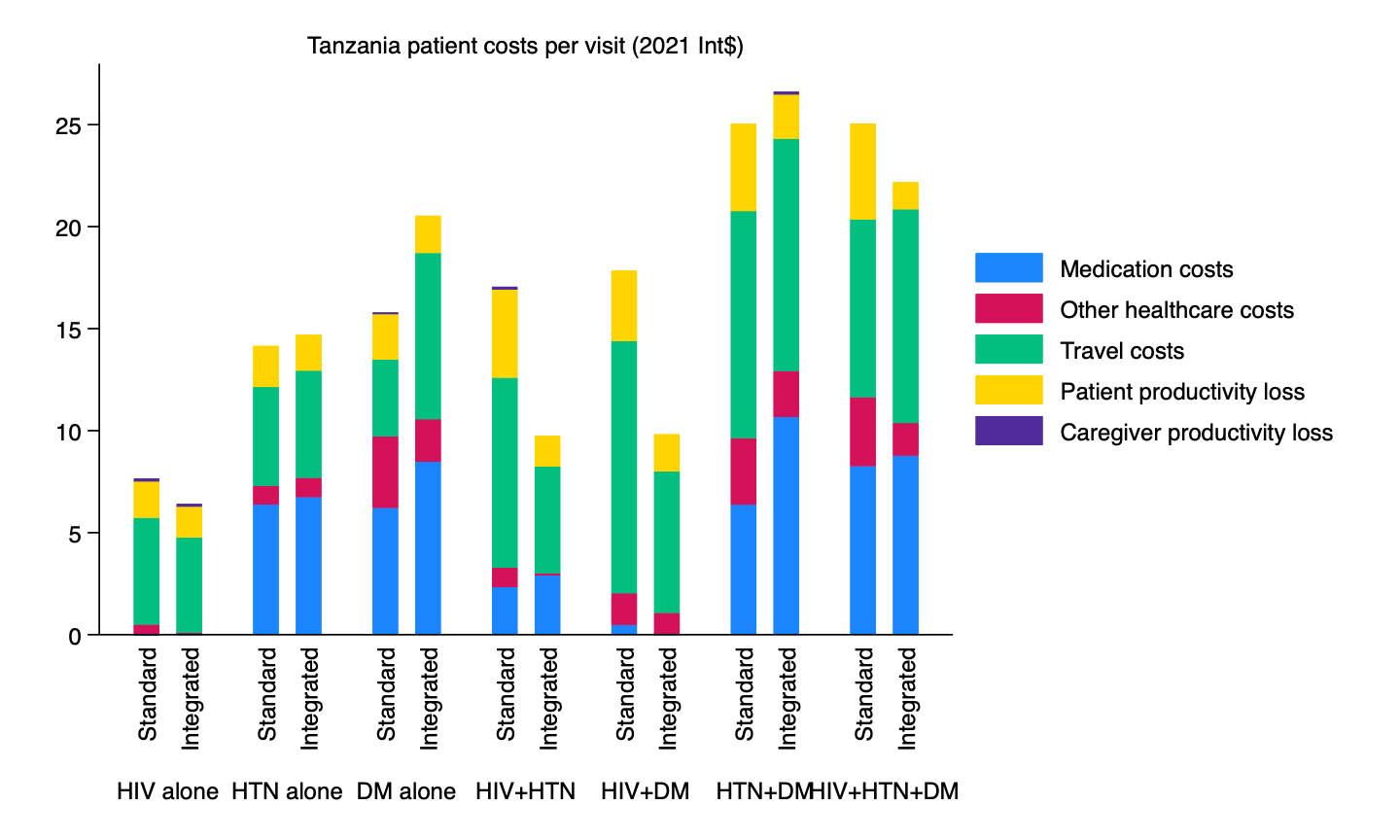
**

**Appendix references:**

1. Shiri T, Birungi J, Garrib AV, Kivuyo SL, Namakoola I, Mghamba J, et al. Patient and health provider costs of integrated HIV, diabetes and hypertension ambulatory health services in low-income settings—an empirical socio-economic cohort study in Tanzania and Uganda. BMC medicine. 2021;19(1):1-15.

2. United Nations Department of Economic and Social Affairs, Population Division. UN Population Division Data Portal: UN Population Division,; 2022 [28 March 2023]. Available from: <https://population.un.org/wpp/>.

3. UNAIDS. AIDSinfo: Global data on HIV epidemiology and response: UNAIDS; 2023 [28 March 2023]. Available from: <https://aidsinfo.unaids.org/>.

4. Zhou B, Carrillo-Larco RM, Danaei G, Riley LM, Paciorek CJ, Stevens GA, et al. Worldwide trends in hypertension prevalence and progress in treatment and control from 1990 to 2019: a pooled analysis of 1201 population-representative studies with 104 million participants. The Lancet. 2021;398(10304):957-80.

5. Internation Diabetes Foundation. IDF Diabetes Atlas 10th Edition. IDF, 2021.

6. United Republic of Tanzania, Ministry of Health, Community Development, Gender, Elderly and Children. Health Sector Strategic Plan July 2021 – June 2026 (HSSP V): Leaving No One Behind. Dar es Salaam, United Republic of Tanzania: 2021.

7. Republic of Uganda Ministry of Health. Republic of Uganda Ministry of Health Strategic Plan 2020/21 - 2024/25. Kampala, Uganda: 2020.

8. Uganda Bureau of Statistics. Uganda National Labour Force Survey - Main Report. Kampala, Uganda 2021.

9. Tanzanian National Bureau of Statistics. Integrated Labour Force Survey (ILFS) 2020/21 – Analytical Report. Dodoma, Tanzania 2021.
